# Views and experiences of compassion in Sri Lankan students: An exploratory qualitative study

**DOI:** 10.1371/journal.pone.0260475

**Published:** 2021-11-24

**Authors:** Lasara Kariyawasam, Margarita Ononaiye, Chris Irons, Lusia Stopa, Sarah E. Kirby

**Affiliations:** 1 Department of Psychology, University of Southampton, Southampton, United Kingdom; 2 Balanced Minds, London Islington, London, United Kingdom; University of Hradec Kralove: Univerzita Hradec Kralove, CZECH REPUBLIC

## Abstract

Practicing compassion has shown to reduce distress and increase emotional well-being in clinical and non-clinical populations. The existing research is primarily focused on Western populations although the concepts of compassion are heavily influenced by Asian Buddhist views. There is a dearth of compassion research conducted particularly in the Asian context. Therefore, this study aimed to explore the views and lived experiences of compassion in Sri Lankan students, to understand whether compassion is a socially embraced construct in Sri Lanka, considering that Sri Lanka is a Buddhist influenced society. Participants’ views and lived experiences of compassion towards themselves and to/from others were also investigated, with a specific focus on their perceived inhibitors and facilitators of compassion. Aims were set to identify whether Western compassion-based practices could be successfully applied to Asian societies such as Sri Lanka. An Interpretative Phenomenological Analysis approach was used to obtain and analyse qualitative data from a convenience sample of 10 Sri Lankan students, recruited from a Psychology course. The phenomenological analysis of the semi-structured face-to-face interviews elicited three predominant themes: *What compassion means to me*, *what I make of it*, and *compassion through facilitators and inhibitors*. The findings suggested that participants shared a similar understanding of the concept of compassion as reflected in the Western definitions. Experiences and views of compassion were shaped by several factors including religion, culture, society, and upbringing. In general, this study revealed that participants were well aware of the concept of compassion as well as its impact on their psychological well-being. Despite this, inhibitors existed in experiencing compassion. The religious and collectivistic-cultural influences need to be further explored and taken into account when implementing Western compassion-based practices to non-Western contexts such as Sri Lanka.

## Introduction

Compassion is defined as a sensitivity to suffering in the self and others, together with a commitment to try to alleviate and prevent it [1]. The term compassion has been discussed for its healing properties for centuries [2], with ancient Latin literature, Buddhist teaching, and Western psychology, all identifying it as a concept related to alleviating suffering [3]. It is also advocated in other religions such as Islam, Hinduism, Christianity, Judaism and Jainism [4]. However, compassion is considered as a key component of Buddhist teaching, and has been actively discussed and practiced in Eastern traditions for many centuries [5]. From a Buddhist perspective, compassion is viewed as an openness to the suffering of others, with a commitment to relieve it [6].

For over two thousand years, Buddhist teaching has emphasised the impact of compassion on dealing with suffering, and facilitating happiness and well-being [7]. Western psychology has also been influenced by Buddhist teaching and implications for psychotherapy for many decades [8]. Indeed, Buddhist practices have been successfully incorporated into positive psychology [9] and the third wave of cognitive behavioural approaches [10], such as Mindfulness-Based Cognitive Therapy (MBCT; [11]), Dialectical Behavioural Therapy (DBT; [12]), and Acceptance and Commitment Therapy (ACT; [13]). Dalai Lama [14], emphasised that happiness is the purpose of life for Buddhists and non-Buddhists, and encouraged contemplative practices of positive psychology, such as mindfulness and compassion for increased happiness and well-being. More recently, within the last two or three decades, Western Psychology has shown a significant interest in the concept of compassion, and have developed compassion-based practices such as Compassion Focused Therapy (CFT: [15]), into psychotherapy.

While compassion is generally understood as a positive emotion [6], evidence suggests that the experience of compassion can sometimes feel unpleasant [7,8]. This nuance is explained by the fact that the conceptualisation of compassion is found to arouse a pleasant feeling [9], whilst the experience of compassion, following exposure to another’s suffering could feel unpleasant [8]. Therefore, people’s subjective views and experiences of compassion are largely taken into account by Western psychology to understand the different emotional reactions caused from compassion [8].

### Theory and practice of compassion

Gilbert, a pioneer of Western compassion research, developed CFT as an integrative, multidisciplinary and process-based approach, underpinned on Buddhist views and several schools of psychotherapy [2,15,16]. CFT was developed to help people with high levels of shame and self-criticism, who were struggling to derive benefits from standard therapeutic interventions. People with pathogenic levels of shame and self-criticism typically come from insecure, negligent or traumatic backgrounds, and often fear seeking compassion and affiliation. This ‘fear of compassion’, is found to be one of the biggest inhibitors to practising compassion towards the self and others [2]. CFT attempts to suppress these inhibitors, and develop compassion using three directional flows, namely, compassion *to others (CTO)*, compassion *from others (CFO)*, and compassion *to the self (self-compassion*: *SC)*.

Western studies integrating CFT and other compassion-based practices have reported psychological benefits, with evident decreases in depression, anxiety, self-harm and several other psychological presentations [17–19]. These practices have also shown a transcultural effect in improving well-being in Eastern Buddhist societies such as Japan [20], Western non-Buddhist communities [21], as well as in Middle Eastern and Muslim societies [22].

### Cultural influence

One’s cultural background plays a vital role in shaping compassion, and these cultural dimensions influence their tendency to show SC and CTO [1]. Research has explored how SC manifests across different cultures. For example, an individual’s functioning, including the extent to which they can develop SC, is congruent with the cultural and societal values they share with their community [23]. Furthermore, Birkett [24] attributed differences between the levels of compassion, to the beliefs and religious practices affiliated to each country, rather than a simple East-West cultural contrast. To date, most compassion studies have been limited to Western countries [25] and even where studies exploring compassion have made cultural comparisons, this has been limited to a few countries [26]. This signifies the need for more diverse cross-cultural research.

As many compassion practices stem from Buddhist principles [27], and Buddhism is mainly practised in Asian traditions, one would expect Asian Buddhist followers to exhibit higher levels of compassion. Furthermore, it appears fair to expect people in Asian interdependent countries where people’s decisions are very much influenced by others in their society, to be more compassionate due to high levels of social interconnectedness, caring, and social conformity shared between one another [26]. However, studies have actually found the opposite result, with people in Asian interdependent societies such as Japan (where Buddhism is practised as a main religion), having higher levels of self-criticism than people in the Western world [28]. In consideration of these findings, Kitayama and Uchida [29] suggested that self-criticism, is often prevalent in emotionally interdependent and densely knit Asian societies. Therefore, levels of SC could be expected to be lower among those in Asian cultures, as self-criticism is a key inhibitor of SC [26]. Social pressure to conform with social norms and values might then hinder one’s SC. Thus, it is important to consider cultural and religious backgrounds, as well as one’s upbringing, into account when understanding the interplay of factors that may impact upon compassion [26].

Sri Lanka is a South Asian multi-ethnic country, where Buddhism is practised by 69% of the population [10,30]. Although the Sri Lankan community is heavily influenced by the teaching of Buddha, the incorporation of Buddhist teaching into psychotherapy and education remain unexploited [10,30] and are yet to be incorporated into the academic curricular of psychological teachings in Sri Lanka [31]. Applying Buddhist practices such as mindfulness and compassion into psychotherapy is also problematic in Sri Lanka, as the lack of knowledge in integrating Buddhist influenced psychotherapy has restricted mental health professionals from accepting such therapeutic methods. This is mainly due to the social view of meditation and Buddhist practices as spiritual practice rather than a psychotherapeutic approach [10].

### Rationale for the present study

With Sri Lanka being a largely Buddhist influenced, collectivistic and interdependent society where social dominance, comparison, and criticism influences one’s identity and behaviour [32], it seems fair to propose that implementing compassion based practices may enhance the well-being of Sri Lankan people. However, while compassion interventions conducted in Asia appear to be minimum, to date, there has been no published research exploring compassion in Sri Lanka from a psychological view, let alone implementing compassion-based practices.

This study, therefore, aimed to explore the views and lived experiences of compassion in Sri Lankan students using the three flows of compassion; SC, CTO, and CFO, with a particular emphasis on their perceived inhibitors and facilitators.

## Method

### Design and participants

The scientific account of compassion greatly depends on the understanding of individuals’ subjective experiences [33]. Thus, a qualitative approach was determined suitable to understand participants’ subjective experiences of compassion in the present study. Although it is critical to conduct qualitative studies to fully understand the challenges that mental health problems pose to experiencing compassion and to uncover potential inhibitors that wider cultural discourses create [34], qualitative research exploring compassion among community populations remain at an infancy stage [34]. Therefore, this study used a qualitative design to gain a realistic and detailed personal account into how Sri Lankan students view and make sense of their experiences with compassion. As IPA provides rich and insightful data, Smith et al. [35] recommend that 4–10 participants are sufficient for a professional study. They also emphasised on the importance of adapting an in-depth semi-structured interview approach as the most suitable method to elicit meaningful qualitative data.

Therefore, 10 Sri Lankan participants (3 males, 7 females), aged between 19–46 years (*M* = 26.1, *SD* = 8.0) were recruited via a purposive sampling method on a first-come first serve basis. Participants’ religious faith varied from Buddhism (*n* = 3), Catholicism (*n* = 1), Christianity (*n* = 2), Hinduism (*n* = 1), Islam (*n* = 2), and Atheism (*n* = 1). They were recruited from an undergraduate psychology course in Sri Lanka and participants declared that they had not learnt about compassion in their course.

### Interview structure

A semi-structured interview guide was created aiming to understand participants’ views of the concepts of compassion and their experiences of SC, CTO, and CFO. Questions also sought to understand participants’ interpretations of the motives behind a person offering and receiving compassion, and the inhibitors and facilitators that participants encountered in doing so. Additionally to the structured interview questions, probing questions were asked when necessary. See Table 1 for a list of questions asked.

**Table 1:** Interview questions.

1. Can you tell me what the term compassion means to you?
2. Can you talk about your understanding of self-compassion? (giving yourself compassion: explain to this if participants are not familiar with the term self-compassion)
3. I would like you to think about one or two occasions when a loved one was going through a tough time or difficult situation. (this could be a family member or a close friend) Can you tell me if you showed compassion towards them? Could you tell me why (or why not)? Can you tell me the things that you did or said to them? What were your feelings and thoughts towards them? And then afterwards…. How did your words and actions affect them? How about you? Was there an impact on you? How did it make you feel? Were there any consequences for you and your life? If the same thing happened again, would you do and say the same things? If so, why/if not, why not? Are there any factors that facilitate or help you to be compassionate towards others? Are there any barriers that make it difficult to be compassionate towards others? *Repeat for CFO and SC accordingly*.

### Procedure

This study was approved by the Ethics committee of the University of Southampton. Participants’ informed consent to partake and the interviews to be audio recorded was obtained prior to starting the study. All names reported in the Results section are pseudonyms used to protect participants’ identity. Pilot interviews were conducted with two participants from the sample, in order to check the feasibility of the interview guide and to observe whether the questions sampled the areas of interest. As the pilot did not show the need to change the questions, interview guide was not refined, and the pilot data were added to the final analysis. Interviews lasted around 20–30 minutes on average.

### Data analysis

All audio-recorded interviews were manually transcribed verbatim, and subjected to Interpretative Phenomenological Analysis (IPA: [35,36]). IPA is a reflexive, transparent approach, which provides a thorough understanding of individual accounts with meaningful interpretations of their relationships to the world and others. The inductive nature of IPA, which is not based on predetermined hypotheses, facilitated the emergence of unpredicted themes [36]. Reid and colleagues [37] emphasised the importance of using IPA in areas that significantly lack previous literature. As the authors were not aware of any psychological research conducted on compassion in Sri Lanka, IPA was considered an appropriate method.

Analysis involved reading the transcripts multiple times to allow the primary researcher to familiarise with the interview content and fully immerse in the narrative. Each transcript contained two wide margins, with the significant meanings written on the left-hand margin and emerging themes on the right-hand margin. Next, the descriptive, linguistic and conceptual comments for quotes were made throughout the transcripts to elicit the themes. Preliminary themes were then developed, amended, and refined. Each theme was summarised and allocated a participant number with an identifiable verbatim quote. In order to confirm the validity of the interpretations, themes were analysed with recurrent reference to the original text and where appropriate, these themes were clustered together based on relevance to originate the superordinate and subordinate themes. This process was repeated until all the superordinate themes and subordinate themes were developed.

## Results

The IPA elicited three superordinate themes (*What compassion means to me*, *what I make of it*, and *compassion through facilitators and inhibitors)* and nine subordinate themes. The first superordinate theme discusses how participants viewed compassion and self-compassion (*sympathetic considerations towards suffering)*, and differentiated the experience of compassion with others versus the self *(self and others*: *it is not the same)*. The second superordinate theme discusses participants’ overall experience of compassion across the three flows, with their perception of the motives behind the compassion received and offered. Subthemes varied based on positive experiences (*positive vibes and genuine motivation)*, negative experiences (*obligations and exhaustion)*, and reluctance to disclose the need for compassion *(disclosure*: *nobody should feel bad about my life)*. The final superordinate theme captures the facilitators *(‘god is good*’: *religion in shaping compassion*, *being there for one another)* and inhibitors *(compassion is conditional*, *society as an inhibitor)* that shaped participants’ experiences of compassion. Themes and subthemes will be discussed in turn and can be found in Table 2.

**Table 2:** Superordinate themes and subordinate themes.

Superordinate Theme	Subordinate Theme
What compassion means to me	*Sympathetic consideration towards suffering* *Self and others*: *It is not the same*
What I make of it	*Positive vibes and genuine motivation* *Obligations and exhaustion* *Disclosure*: *Nobody should feel bad about my life*
Compassion through facilitators and inhibitors	*‘God is good*’: *Religion in shaping compassion* *Being there for one another* *Compassion is conditional* *Society as an inhibitor*

### Superordinate theme: What compassion means to me

Participants discussed their understanding about compassion and what it meant to them. Compassion was needed the most in tough times and participants viewed it as an innate ability to express a sympathetic consideration towards one’s suffering. However, many participants found it easier to show CTO than towards themselves. Some participants also found accepting CFO challenging. This superordinate theme elicited two subordinate themes: *sympathetic consideration towards suffering*, and *self and others*: *it is not the same*.

#### Subordinate theme: Sympathetic consideration towards suffering

This theme discusses how participants viewed and defined compassion and self-compassion. Most participants conceptualised giving CTO, when describing what compassion is. However, they recognised that the term self-compassion refers to the compassion that is given to the self. All participants had a similar view of what compassion is, and explained that compassion meant being actively engaged in one’s life by showing a level of understanding, kindness and love, particularly in tough times.

*‘It*’*s being kind to people*, *being understanding*, *kind of understanding what they are going through and feeling sympathetic about their situation*’.

(Angelo, aged thirty-two years).

Mathew believed that having a sympathetic consideration was not enough and that one should make an effort to go out of their way to relieve the suffering of another.

*‘My family and I*, *we always tell it to put ourselves in other people*’*s shoes and even if we have to go out of our way to help them*, *that*’*s what compassion is to me*’.

(Mathew, aged twenty-six years).

Many participants discussed self-compassion as the love for oneself and taking care of the self with kindness and the understanding that problems are a shared human experience. Hafsa described the process of generating SC as firstly, identifying who you are, and what you want with life, and then acting on that self-reflection to create a stronger version of herself, amidst suffering.

*‘Me being compassionate to myself is me being understanding in my own self*, *when I*’*m in a really bad situation*, *trying to bring myself up through worse*, *it*’*s like talking to a mirror*. *I want to keep myself strong*, *keep myself happy*, *I know what I want*’.

(Hafsa, aged twenty-one years).

SC was also seen as a process, which takes time and effort to attain.

*‘My understanding is it is a process*. *It is basically a goal that needs to be reached through a process where you are able to show love and care towards yourself*. *But it doesn*’*t happen overnight*. *And you need to work towards it and be aware of what your needs are and what who you are to be compassionate towards yourself*’.

(Radhi, aged twenty-four years).

#### Subordinate theme: Self and others: It is not the same

Although all participants acknowledged the benefits of SC, most of them struggled to experience it. Even though they showed CTO, they seemed judgemental and particularly harsh towards themselves when they were going through a difficult time themselves. When questioned about this disparity between CTO and SC, participants explained that the experience of SC was not the same as showing CTO, or even receiving CFO.

*‘Helping another person comes easier to me than helping myself because you feel a lot of sympathy when you see something bad happening to someone else*. *That same level of sympathy is very difficult to have towards yourself when you are in a difficult situation*. *You tend to be more critical and feel a lot of guilt*. *Your personal judgements about the way you acted in that situation and the guilt you have and the part that you have landed yourself in prevents you from sympathising towards yourself*. *That makes it difficult for you to help yourself because you are not putting yourself in the victim shoes*. *So*, *you are seeing yourself responsible*, *you don*’*t really feel like you deserve the help*. *It is difficult to feel bad about yourself and try to help yourself*’.

(Angelo, aged thirty-two years).

Radhi added that people set themselves high expectations and when these are not achieved, they beat themselves with a sense of harsh self-criticism and judgement.

*‘I think we always put ourselves in a box where we are so protected and guarded but then we want each and every one of our efforts to be impressed or acknowledged or recognised*. *We do not give that space to ourselves as much as we do for others*. *I think we are harsh on ourselves and judge ourselves harder than we judge others*’.

(Radhi, aged twenty-four years).

### Superordinate theme: What I make of it

This superordinate theme discusses participants’ overall experience of engaging in the three flows of compassion. To many, this was a healing and positive experience, which motivated them to continue seeking and offering compassion, whereas for others, the experience was rather negative. Experience was mostly positive when participants viewed the compassion received and given as genuine, whereas the compassion perceived as fake or obligatory was experienced negatively, causing exhaustion.

#### Subordinate theme: Positive vibes and genuine motivation

All participants recollected memories where they showed compassion to someone they cared about and memories where they received compassion from a loved one. While participants found it easier to engage compassionately with people they knew well, the overall experience was positive when they offered compassion because they genuinely wanted to alleviate the suffering of the other, as well as when they received compassion from someone who genuinely wanted to help.

*‘At the end of the day*, *I knew that if I did not have them in my life*, *I would not have thought of this solution and my situation would have been much worse*’.

(Angelo, aged thirty-two years).

*‘Their actions*, *words helped me a lot*. *My sadness and tension reduced*. *I could be the happy girl they wanted me to be*. *It didn*’*t take so many days for me to recover*’.

(Ashini, aged twenty years).

While most participants felt positively about receiving CFO, they believed that the feeling was mutual to those who showed them compassion.

*‘I*’*m sure they felt good about themselves too*. *They*’*ve been through similar issues*. *More than helping me*, *they were honestly doing themselves a favour*’.

(Sonali, aged twenty-five years).

In addition to feeling good about receiving CFO, many participants found the experience of offering CTO rewarding and self-satisfying.

*‘I feel whenever I become compassionate to someone*, *I feel like this vibe going out of me*. *I feel good when I show love to someone*, *it*’*s a self-satisfying thing for me*’.

(Ashini, aged twenty years).

#### Subordinate theme: Obligations and exhaustion

It was striking that although most participants believed that they genuinely expressed CTO, they questioned how genuine others were, when offering compassion. While some of them viewed compassion they received as genuine, others perceived it as an obligation or an artificial expression of care. When compassion offered/received was perceived as fake or obligatory, the overall experience of the compassionate engagement was felt exhaustive and negative.

*‘That*’*s a fake thing*. *I feel that*. *They say oh just ignore that*, *you*’*re a good person*, *don*’*t be too emotional*, *when we*’*re in a situation you*’*re the one who help us*, *they say so*. *But I think it*’*s because I help them*, *not because they want me to be happy*’.

(Heshan, aged twenty-seven years).

Nelu explained that offering compassion is not always an easy or pleasant experience, and that when it had to be done out of obligatory reasons, she felt exhausted and tried to distance herself from others in order to avoid giving CTO.

*‘Sometimes it*’*s actually being a little distancing as well because they keep coming back to me and with my work*, *I feel distracting*. *To tell the truth*, *I have been irritated*’.

(Nelu, aged forty-six years).

Sonali too had a very different, yet an excruciatingly painful experience from having shown CTO. This was because she was too emotionally involved in the other person’s suffering to a point that it became detrimental to her own well-being.

*‘I was very emotionally down*. *It*’*s a memory that I will never be able to erase from my mind because it*’*s not me who went through it*, *it*’*s that particular person*, *but I felt really depressed*, *down and I didn*’*t have an appetite for a couple of days*. *I felt really helpless and I honestly felt that there was no purpose of living*’.

(Sonali, aged twenty-five years).

#### Subordinate theme: Disclosure: Nobody should feel bad about my life

Whether the overall experience of receiving compassion was negative or positive, all participants found comfort in knowing that they received CFO and that they were not alone in their suffering. However, some of them were reluctant to seek compassion and disclose their problems to others, which eventually inhibited them from receiving CFO.

*‘I don*’*t show that I am down*. *I*’*ve always been down in a way*, *but I don*’*t want others to feel down because of me*. *I never want to add to anyone*’*s problems*’.

(Fatima, aged nineteen years).

To Mathew, because of his previous negative experiences of seeking CFO, suffering alone was easier than disclosing his struggles or putting his parents through pain.

*‘Depression and overweight don*’*t help*. *I*’*m not the one to show it*. *For me to even tell my mom that I was going through depression was huge*. *I don*’*t tell anyone*’.

(Mathew, aged twenty-six years).

### Superordinate theme: Compassion through facilitators and inhibitors

Participants also discussed what factors they believed facilitated their experiences of compassion and the factors that inhibited it. Most participants signified the importance of their religion and culture in shaping compassion. Some of them however, elucidated how one’s religion and culture could restrict their compassion only to those who belong to the same religion and culture, and could act as an inhibitor towards showing CTO who belong to out-groups. On the other hand, all participants indicted society and the stigma surrounding certain social constructs and norms as their biggest inhibitor to compassion.

#### Subordinate theme: ‘God is good’: Religion in shaping compassion

Participants of this study were from various religious backgrounds. Despite this variety, most of them discussed the role their religion played in shaping their compassion.

*‘So*, *in my religion which is Christianity*, *we are always taught that we should show compassion to others and it is something that you grow with*. *It is not something you should do*, *but something you should get from within you*’.

(Ashini, aged twenty years).

Fatima recalled how God protected and prevented her from pleading help from others.

*‘Whenever I ask my god for something*, *he has created me not to go and beg or cry for someone*. *He is there with me*, *so why do I need other people in my life*?’

(Fatima, aged nineteen years).

Nelu, a Buddhist follower explained how she incorporated mindful meditation into practicing compassion.

*‘I learned about compassion through meditation*. *I*’*ve been doing this Buddhist meditation for the past 10 years*. *It gives you a deep understanding of the things in mind and matter*. *So*, *you*’*re able to analyse and understand things that*, *it*’*s happening because of this and what needs to be changed*, *and if it cannot be changed*, *you have to just accept it*. *Most of the time it*’*s all about accepting*, *accepting the present moment and living in the present moment*, *going step by step and going with the flow*’.

(Nelu, aged forty-six years).

#### Subordinate theme: Being there for one another

Participants also discussed how the collectivistic social dynamic in Sri Lanka, reminds them that they are not alone in their suffering. Angelo viewed the Sri Lankan culture as a close-knit entity, which postulates that for the society to move forward as one, people need to show love and kindness to one another.

*‘Culture says that for our society to survive*, *to come to a better place*, *we need to be there for each other*. *Our culture is a communal one where people tend to look into other peoples*’ *worries and difficulties*, *and stick to like family*. *And even the extended families and friends and relations and everyone are closely tight together*’.

(Angelo, aged thirty-two years).

Nelu emphasised how being brought up in a cultural and religious background shaped her to become the compassionate person that she is.

*‘I have values*. *When I*’*m compassionate towards others*, *I use them properly and also being in a very religious*, *we are from a family very helpful*, *religious and cultural background*, *so I think those things also mattered in who I am*’.

(Nelu, aged forty-six years).

#### Subordinate theme: Compassion is conditional

This theme stands out from the rest as it describes a completely different aspect of how culture and religion could affect compassion. Some participants pointed out how the elements that others described as facilitators of compassion, could at the same time act as inhibitors towards the emergence of compassion when seen from a bigger picture.

*‘If I talk about the culture that I am living in*, *people are compassionate*, *but if you start comparing this culture and another culture*, *it*’*s definitely different*. *They are only compassionate towards people who share their same beliefs and who are in their same belief system*. *But when that changes a little bit*, *you are either from a different religion*, *or different racial background or different educational or socio-economical background*, *that compassion changes*. *So obviously this compassion is very conditional towards the person*’*s background*. *That*’*s how I see culture has influenced this society*. *Therefore*, *having been religious before*, *has actually taught me how I can be compassionate even without religion or the teachings of a religion*’.

(Radhi, aged twenty-four years).

Mathew, who spent his childhood in Australia, further supported Radhi’s allegation. Mathew described how upon return to Sri Lanka, the way he was treated made him feel like an outsider, and how this experience affected him negatively.

*‘Coming from a country (Australia) where decency and manners are the most important and coming to a country like this (Sri Lanka) where people don*’*t even realise it*’*s missing*, *I felt helpless*. *I said I felt like a foreigner in the country I was born*. *I feel like that now*. *Nobody understands unless you have been through the same thing*’.

(Mathew, aged twenty-six years).

#### Subordinate theme: Society as an inhibitor

Participants’ experiences of being judged or criticised by the society for offering CTO, led them to fearing or being discouraged to genuinely show CTO. Heshan attributed social judgements to the ‘tradition’, as he emphasised that people become narrow minded by being stuck within this outdated framework. Heshan’s statement tallies with what Radhi and Mathew described earlier as compassion being conditional from one entity such as tradition to another.

*‘In Sri Lanka*, *when I try to help a girl*, *people see it as a different thing*. *My parents too*. *There are so many friends who comes to me when they need help*, *mostly girls*. *My parents sometimes misunderstand that I have many girlfriends*. *But that*’*s not true*. *I want to help them*. *And if we consider about other people*, *I mean the society*, *they see it as a real different thing*. *Because they are in a frame called tradition*’.

(Heshan, aged twenty-seven years).

Showing CTO resulted in Fatima losing her own support system. She was even condemned by other people, when she was treating her own self with compassion.

*‘People call me overconfident when I*’*m self-compassionate*. *That affected me*. *When I helped my friend*, *they were like you*’*re not the godmother to go and explain people and make them understand*, *why do you have to worry about them*? *Many people blame me for supporting her*. *Many people ignored me*, *my best friend totally ignored me*, *she*’*s not even talking to me and that affected me a lot and still it does*’.

(Fatima, aged nineteen years).

Fatima was not alone in feeling discouraged to give herself compassion due to social judgements. In addition to being discouraged to show CTO, Radhi too found it difficult to show SC when people judged her. She stressed how she internalised other people’s negative attributes.

*‘When others are mean or judgemental to you it*’*s difficult for you to be compassionate towards yourself*. *You take other peoples*’ *views into account of how you should treat yourself*. *When somebody is mean to you when you*’*re sad*, *you start thinking ok maybe it*’*s my fault*, *maybe what I did was so wrong that I cannot forgive myself*’.

(Radhi, aged twenty-four years).

The discussions implied that participants believed lack of awareness fuelled narrower views and social judgements. For instance, participants emphasised that people were more judgemental when their knowledge of mental illness was limited. Mathew described how disclosing his struggles with depression to his mother further disappointed him. However, it was intriguing that instead of feeling frustrated, Mathew expressed a great sense of compassion towards his mother.

*‘I fell into this depression where I felt like a rain cloud was following me everywhere*. *I told my mom I*’*m feeling like this*. *But they are old school*, *born in the 50*’*s in an era when people told to just get over it*. *So*, *I told her this is what I was going through and she said why don*’*t you just get over it*. *I know your normal reaction is angry*. *I understood*, *I understand if I grew up in that era*. *Life is easier for us these days*, *they had to go through a lot and they just got over it*, *they had to*’.

(Mathew, aged twenty-six years).

Similar to this experience, Sonali explained that her father was not being understanding of her mental health struggles. Again, however, she too expressed a sense of understanding towards her father’s reaction, as she felt that the Sri Lankan society in general lacked understanding of mental health problems and people just do not know how to help.

*‘I was feeling very down to a point that I felt no purpose of living anymore*. *If my father was understanding*, *I would have overcome way easier*. *I don*’*t blame him*. *He*’*s not a compassionate person*. *But because of the way this society is*, *people of his generation don*’*t understand these struggles*. *In Sri Lanka*, *people don*’*t really know much about mental health*. *They just think people with depression are weak and don*’*t know how to help*. *Instead*, *they just hide mental illnesses to just avoid judgements*’.

(Sonali, aged twenty-five years).

Insecure relationships and lack of reassurance from parents also inhibited participants’ experiences of compassion.

*‘My father is not a compassionate person I would say*. *So*, *when I talk about my father that brings up you know my whole history from the time I remember up to now*’.

(Sonali, aged twenty-five years).

*‘I barely appreciate myself*. *Because even if I do something great*, *my parents don*’*t appreciate me*. *So now*, *I don*’*t appreciate anything*. *I have difficult parents who worry about what others think*, *so they got me engaged to a guy who I don*’*t know*’.

(Fatima, aged nineteen years).

These comments greatly imply that society and significant others have a major impact on participants’ compassionate engagement. These will be further discussed in relation to existing literature in the Discussion section.

## Discussion

This study aimed to explore and understand Sri Lankan undergraduate students’ views and lived experiences of the concept of compassion within the three flows: SC, CTO, and CFO. The objective was also to understand perceived inhibitors and facilitators that Sri Lankan students encounter when expressing and experiencing compassion. Findings will be discussed in relation to their corresponding themes.

### What compassion means to me

Compassion is known as a sensitivity to suffering in oneself and others, with a motivation to alleviate and prevent it [1]. Gilbert [38,39] outlined some key competencies involved in compassion, including the motivation to care, tolerance of negative emotions, sympathetic concerns, and non-judgemental and empathetic understanding. Although participants had not previously learnt about compassion in their psychology course, everyone shared a similar view to Western definitions whilst actively recalling memories of previous compassionate engagements. Their recalled experiences with compassion implied that they were well aware of its impact on increasing well-being and reducing suffering. Participants described compassion as not just being sensitive towards suffering, but also having the motivation to relieve that suffering. When asked to describe their understanding of compassion as a construct, they shared a similar understanding to Western explanations, and expressed genuine sympathetic considerations towards people’s problems, motivation to alleviate suffering, and kind and loving feelings as attributes of compassion. Compassion is viewed as stepping out of one’s typical frame of position and perceiving the world from a standpoint of another [40]. In support, many participants identified the act of showing compassion as a process that requires not only sympathetic considerations towards suffering, but also a conscious effort to achieve.

Participants then described self-compassion as compassion given towards the self. Their views of SC was in line with that of Neff [41], who defined SC as being kind and non-judgmental towards oneself and their own suffering or failure, while intending to alleviate that suffering rather than being harsh towards the self or avoidant of that suffering. Overall, it appeared that despite the absolute lack of compassion research conducted in Sri Lanka, interviews of the present study indicated that Sri Lankan students would possibly benefit from compassion-based practices as they shared a good knowledge of compassion as a construct and of its benefits in increasing their psychological well-being.

### What I make of it?

Despite being fully aware of the concept and its benefits, most participants however reported feeling as if they struggled to develop SC although they found it easier to give CTO. To many, ‘guilt’ obstructed SC and incited self-blame and criticism instead. Although many psychological models emphasise that humans are primarily guided on self-interest [42], studies signify that humans are more likely to use harsh language towards themselves rather than to a loved one, or even a stranger in that regard [41,43]. Participants’ statements such as ‘*I*’*m wasting my life*’ (Mathew), and *‘I was ugly and fat*’ (Fatima) further confirmed this. Furthermore, studies emphasise that some people who are extremely compassionate to others have a tendency to be harsh towards themselves even when things are out of their control [41]. People generally have a different way of viewing others when compared to how they view themselves [19] and become self-critical when they fail to achieve what they want, self-inflicting pain [44]. On the other hand, studies have found that females have slightly lower SC and higher CTO than men [45], due to females’ natural propensity towards nurturing and compassionate care than men [46]. This gender difference may have also affected the present study, as 7 out of 10 participants were females. Further quantitative and mixed method studies could be conducted to explore the possible differences of gender, age and other demographic factors on the varying levels of compassion.

Participants also enjoyed giving CTO and receiving CFO when they believed it was offered genuinely. In the interviews, they expressed that they could sense when someone was being real, and recognised when the compassion felt superficial. In such cases, it appeared to participants that the compassion that was offered, was done so with a sense of obligation, and was perceived as forced. Heshan explained *“I think it*’*s because I help them*, *not because they want me to be happy”*, igniting a powerful understanding of the true motive behind CFO. From an evolutionary perspective, compassion is not always seen as unconditional due to its propensity towards cost-benefit outcomes [47]. The reciprocity norm [48] suggests that people generally feel obliged to reciprocate help when they have received favours from others. This transactional behaviour raises a question for future research to consider, with regards to how genuine this type of compassionate giving is, or if people who feel obliged to offer CTO really want to relieve the suffering of others. Catarino and colleagues [49] attributed this perceived difference to ‘genuine compassion’, which is the genuine concern for others’ needs and the motivation to help them [2], and ‘submissive compassion’, the caring that develops for self-advancing or defensive needs in order to be liked by others or to avoid rejection. In this study, most participants felt genuine compassion for others, while they implied that the compassion they received from some people might have been submissive.

However, when the motivation was genuine, participants believed that it benefited both the recipient and the giver of compassion. They stressed that people who had experienced similar life events could find comfort in offering CTO. This is consistent with previous research. For example, Catarino et al. [49] established that giving CTO positively influences one’s own well-being. Studies have also found positive relationships between CTO and life-satisfaction [37], prosocial behaviour [40], self-esteem, self-awareness, and negative relationships between CTO and depression [49]. In addition, the phenomena of giving CTO have led to increased feelings of connectedness, social support and trust [50].

Whilst many participants found the experience of receiving and offering compassion to be pleasant, this was not the outcome for some others. Nelu felt exhausted from showing CTO, although she felt compelled to do this. Fears, blocks and resistances of compassion [51] can often hinder compassion. Being overwhelmed by distress can generate a sense of fear, while lack of understanding of compassion or environmental and external difficulties can lead to blocks. People also resist compassion when they do not want to be compassionate due to exhaustion, previous negative experiences and for other reasons. This implies that compassion offered with obligation (submissive compassion) may not feel as satisfactory as genuine compassion, which would then be reflected in the experience of the recipient.

Despite the perceived benefits, compassion can lead to avoidance or fear reactions in some individuals, especially among those with high self-criticism [15]. Gilbert [15] found that for some people, the experience associated with CFO can generate grief feelings of wanting but not getting the love and care from loved ones, which can increase the awareness of an inner loneliness. In such situations therefore, if the experience of receiving CFO is unfamiliar, people would dissociate or avoid the CFO. CFO is an under-researched area and, it would be interesting for future research to explore whether the discrepancy between believing participants’ own compassion to be genuine and CFO to be submissive, is due to participants’ own fear reactions, and if this was their reason for avoiding CFO.

In support, Fatima and Mathew discussed how maintaining a negative self-image pushed them away from seeking CFO. Most participants exhibited problems with overthinking, anxiety and rumination, implying a link between these traits and low self-compassion. Overthinking, anxiety and rumination lead to low self-esteem, which in turn guides people to be their own worst self-critics [52]. This is supported by previous cross-cultural research, which emphasised that people in Asian collectivistic backgrounds indicated higher self-criticism and rumination than those in the West [53,54]. Self-criticism in collectivistic cultures is considered as a facilitator of self-improvement that helps to perpetuate social harmony [55,56]. Thus, participants’ experiences with low SC and reluctance to seek CFO might have had a cultural impact.

### Compassion through facilitators and inhibitors

Although experiences and recollections of memories varied, all participants shared a set of factors that enabled their compassion and others that hindered it. Despite participants following different religions, almost everyone believed that religion taught them to be compassionate. Studies have found that religion plays a major role in the majority of the Sri Lankan society especially when determining self-identity [57]. This is consistent with other studies that religious identification is the strongest constituent of one’s self-identity [58]. Although compassion is central to the Buddhist teaching, studies have found that compassion is taught and practiced in many other religions, including Christianity [59] and Islam [22]. Deliberating on the links between compassion in Buddhism and Islam, Shah-Kazemi [60] demonstrated that compassion is inseparable from love in both religions and that the level of loving compassion defines the core of one’s humanity. Successful application of compassionate practices among non-Buddhist, Western communalities [26], and Middle Eastern Muslim countries [22], imply the transcultural applicability of compassion practice.

All participants discussed the idea of a collectivistic culture that encourages its members to be there for one another during tough times. These cultures and their values often shape human behaviours and thought processes [61]. Values are socially accepted concepts that are set to help achieve motivational goals such as well-being and social interaction among groups [62]. Tradition is one of these values that requires obligation, respect and acceptance of the norms that one’s culture or religion postulates. Gilbert et al. [63] predicted that cultural elements such as social norms might influence SC, while Neff et al. [26] suggested that SC might be at least partially culturally determined. Particularly, people in Asian, Buddhist influenced societies may practice compassion more naturally, given that SC and CTO is central in the Buddhist worldview [26]. In fact, in Buddhist societies such as Thailand, failure is seen as an opportunity for self-improvement, while Buddhist compassion has influenced ethics of daily living such as child rearing [64].

People are more likely to show compassion towards those who share similar values, beliefs, preferences, actions and physical characteristics with them [65]. According to evolutionary theories, compassion is subjective to evolutionary demands, leading people to be more compassionate towards kin and possible reciprocators of compassion than to those with whom they are unacquainted and non-kin [66]. Although culture helps people identify and unite within their social group, they can encourage people to negatively evaluate those who do not comply with these values [62]. Radhi, a participant of the present study identified this disparity and explained how society, culture and religion could inhibit compassion as opposed to being a facilitator. She further emphasised how these entities manipulate the level of compassion one should show to others, based on the in-group outgroup diversion. By forming groups and societies, social dominance leads people to identify their groups as superior to other groups and, as a result, act in non-compassionate ways to outgroups [67]. Furthermore, obedience to authority, having to abide by group norms and the pressure of fitting in have diminished people’s ability to show CTO [68].

Contrary to the aforementioned Asian Buddhist influence on compassion, some studies have found that people in such collectivistic societies exhibit a narrower trust radius, meaning a narrower width of the circle of people they trust, when compared to people in individualistic societies. This is due to collectivistic societies being more discriminatory and limiting of trust towards those in an out-group when compared to those in an in-group [69]. The lack of trust may also explain why participants viewed society as an inhibitor towards experiencing CTO as well as CFO. As previously detailed, the very definition of tradition itself highlights the need for ‘obligation’ and, Heshan in his interview emphasised how Sri Lankans are limited in their understanding and acceptance of people due to the ‘*so called frame of tradition*’. Participants’ understanding of this disparity raises the question whether culture and religion actually influence people to be more compassionate or, whether they hinder one’s compassion, signifying the void for further exploratory research.

Another inhibitor participants identified was the stigma and lack of awareness in the Sri Lankan society surrounding the topic of mental illness. Mathew emphasised how his mother wanted him to *‘just get over*’ his depression, when he disclosed his struggles with depression to his mother. Many Sri Lankan families try to hide mental illness of family members from the society to avoid stigma and discrimination, as mental illness is seen to restrict peoples’ chances at employment and marriage [70]. Fear of stigma was seen as the biggest obstacles to seeking professional help and, even when help was sought, studies indicated that there is minimum reintegration into families following psychiatric treatment in Sri Lanka [71]. Negative stigmatising views from others could create self-stigmatising thoughts in people, which consequently result in social withdrawal [61]. Such individuals become increasingly sensitive to threat and less adherent to self-soothing actions [27]. In the present study, Sonali described how society views depression as *‘being weak*’ leading people to conceal mental illnesses. Studies also showed that Sri Lankan children are raised to fear shame and are ridiculed by the society if social norms such as norms of sexual modesty and approved behaviour are subverted. Fear of being ridiculed by the society was evident in this study as participants discussed being called overconfident and criticised for showing SC and CTO.

Consequences were a lot more severe when participants were misunderstood or judged by their own family, especially parents. Gilbert [38] emphasised that the attachment system from significant others acts as a foundation for developing capacities for compassion. Thus, the lack of care in early relationships may lead people to feel underserving of love and fear compassion [63]. Fear of compassion manifests in negative self-evaluations resulting in detachment from society [41]. In the current study, participants with negative self-perceptions and weak attachments with significant others were reluctant to seek help, or, according to them, ‘be a burden’ on others. In a non-compassionate self-perception, people view themselves as underserving and others as deserving of compassion, creating a fabricated sense of separation from the rest of humankind. Self-critics often feel inadequate and believe that they are the only ones facing failure. This irrational belief that everyone else is perfect, makes them isolate themselves from others [44]. This is one of the main challenges for psychotherapy, as people fearful of compassion tend to be avoidant or not disclose personal struggles hoping to escape receiving CFO [39]. Thus, it is apparent that participants’ self-criticism may have stemmed from unappreciative, insincere significant others, in this case, parents and family.

Taken together, findings indicate that Sri Lankan students’ views and lived experiences of compassion reflect those discussed in the Western psychological models. These findings are not entirely surprising due to compassion-based practices such as the Compassion Focused Therapy [39], being at least partly influenced by Eastern and Buddhist practices. Findings therefore, suggest that the Western therapeutic models and practices of cultivating compassion and SC, along with the recognition of fears, blocks and resistances may also be of use to the Sri Lankan population.

#### Strengths and limitations

This study was the first psychological entrance to investigate compassion in a Sri Lankan sample. The study sample also represented a variety of religions that are presently practiced in Sri Lanka. Use of IPA provided a deeper understanding of participants’ lived experiences of compassion as well as their perceived inhibitors and facilitators of compassionate engagement. In addition, the present study indicated that psychology students, even though they had not previously learnt about compassion, were familiar with the construct and were appreciative of its impact on their well-being. This suggests that Buddhist practices such as compassion practice could be incorporated into the academic curricular of psychology courses. However, it is also important to remember that all participants were psychology undergraduates, who may have had an above average understanding of the concept of compassion, and that social desirability bias may have played a part in all of them disclosing having shown CTO. Thus, future research could seek to replicate this study in a non-psychology student population.

Although the small sample size meant that generalisation of the findings is limited, sufficient information about participants’ experiences was obtained. In fact, IPA supports the use of small samples to extract in-depth rich data and emphasises that small samples can generate considerable accounts of individuals’ perceptions [36,72]. However, the use of undergraduate students may not be generalised to other participant groups although qualitative research is not designed to represent entire populations, but rather to deliver in-depth intuitions into chosen matters [73]. Notwithstanding the limitations, this study provided an important insight into the role that cultural factors such as religion and society play in shaping one’s experiences of compassion towards the self, to/from others. These insights can be used in future research to explore the representativeness and generalisability in quantitative studies with larger sample sizes.

## Implications for future research

An overarching aim of this study was to provide a knowledge base that could contribute to the development of prospective compassion interventions. Findings indicated that Sri Lankan students’ views of compassion and its impact is in line with Western studies. However, there is an apparent need for a deep investigation into cultural elements such as social norms and how these interplay with fear reactions to inhibit compassion as well as protective factors such as religion and significant others towards facilitating compassion. Such investigations would contribute to the successful application of Western compassion practices, to collectivistic societies such as Sri Lanka. Furthermore, prospective studies may incorporate psychoeducation to increase awareness of mental health problems and to promote acceptance and help-seeking behaviour in the Sri Lankan community.

## Conclusion

This study investigated Sri Lankan undergraduate students’ views and lived experiences of the concept of compassion, with a specific focus on their perceived inhibitors and facilitators. Findings suggest that Sri Lankan students are well aware of the concept and meaning of compassion, and have experienced compassion in all three forms; *SC*, *CTO*, *and CFO*, to different extents. Whilst all participants acknowledged the benefits of receiving and offering compassion, some of them found the experience of offering compassion to be exhausting or unpleasant. They also questioned the genuineness of the motives of others, although they still found comfort from receiving CFO, regardless of the perceived motives. Despite acknowledging the powerful impacts of receiving compassion, those with insecure attachments with others especially parents, were hesitant to seek CFO. Most participants believed that religion and cultural upbringing shaped their compassion, while society in the forms of judgements, discrimination and stigma inhibited their compassion experiences. This study, therefore, suggests that clinicians should consider these culture-specific factors when implementing compassion-based practices in Sri Lankan people. There is however, also the issue of stigma surrounding mental illness in this community that may act as an inhibitor to seeking treatment, which will need to be carefully considered in the planning of interventions aimed at developing a more compassionate approach.

## Supporting information

S1 File Interview transcripts. (PDF)
