# Supplementary material for: Views and experiences of compassion in Sri Lankan students: An exploratory qualitative study
Source: PLoS One. 2021 Nov 24;16(11):e0260475. doi: 10.1371/journal.pone.0260475 (PMC8612518; doi:10.1371/journal.pone.0260475)
Supplement: S1 File — (PDF) [file pone.0260475.s001.pdf]

Interview Transcripts

I: Interviewer

P: Participant

Interview 1.

I. So, the first question. Can you tell me what the term compassion means to you?

P. Mmm it’s being kind to people, being understanding, kind of understanding what they are going through and feeling sympathetic about their situation

I. Hmm, ok brilliant, so you don’t necessarily need to give me an you know an exact definition, umm anything that comes to your mind. Anything else you want to you you can think of?

P. No that I guess that that is pretty much the answer I wanted to give

I. Ok. Second one um Can you same thing but can you talk about your understanding of the term self-compassion?

P. Ummm I guess it is being understanding about yourself and understanding what you are going through and kind of taking care of yourself and tending to like personal needs like both physical and emotional I guess

I. ok umm so the next set of questions are going to focus on three aspects of compassion. The first one is how we show compassion to other people and second is how others show compassion to us and umm the third is how we show compassion to ourselves.

P. Ok

I. ok? So for the first one, Id like you to think about one or two occasions when a loved one was going through a tough time or a difficult situation. This could be a family member or a close friend and could be an event where they were struggling and needed help.

P. okay

I. do you need time to think of any any situation or do you already have?

P. No I have a few memories like that in mind so I think I can proceed

I. Brilliant. So my first is can you tell me if you showed compassion towards them?

P. Yes I think so,

I. Ok could you tell me why

P. Ok so, it was it was a difficult time for them so what I did was I spoke to them you know I kind of listened to their pain of what they were going through and kind of shared in their pain. So I think I kind of helped them unburden some of the things that they were going through in the first place. And then when they there were occasions when they said they need this specific favour in order for them to you know get through this tough situation and I obliged and I kind of helped them with that as well in in a way that was possible for me to do so. So I think all in all I was able to show compassion to them when they were needed that.

I. So can you tell me the things that you did and said to them that you think were compassionate towards them

P. Umm things like you know umm I think I can't begin to comprehend what you are going through I don't think I can really you know even comprehend the pain that you are going through and you know if you want anything I'm here you know I'll see what I can do. And.. Yeah statements like that I guess covered how I would respond and how I did indeed respond in a situation like that

I. So it was all the things that you said to them that you think were

P. Ok there were specific actions that I did as well,, but

I. Could you tell me what?

P. Ummm for an example.. so they said that you know they needed to go out basically to you know get their mind off of the problem that they were going through.

I. Okay

P. So you know we made it a point to go for a dinner you know or go for an outing so they would be distracted and you know they'd forget about their problems at least for that moment.

I. Mmhmm

P. yeah

I. Wonderful. So, what were your feelings and thoughts towards them?

P. I felt you know sympathy I guess and (unclear). Those are difficult things to go through and not like anything anyone would wanna go through ideally. So seeing them in their situation and you know seeing that they can't immediately overcome their problem or their that you know get them selves to a better position. That feels its very sad thing you know from my perspective to see one of my friends or a person I care went through that.

I. So would you say you felt sad and sorry for them?

P. Yes, Of course

I. Okay. And then afterwards…. How did your words and actions affect them?

How do you think they affected them?

P. I think you know umm at least there were a few occasions it did help.

I. Mmhmm

P. I'm not saying it completely changed the outlook of their problem or the situation that they were going through

I. Mmhmm

P. But it helped at least to a certain level where they felt you know slightly better than what they were beforehand

I. Ok, How about you? Was there any impact on you?

P. I honestly I feel that a little bit better about myself you know being able to help someone like that. Its always very rewarding when you can do that for someone. especially for a person that you really care about

I. Yeah

P. So

I. Okay. If the same thing happened again, would you do and say the same things? Or would you do anything differently?

P. Umm I just you know. Um I would actually try to speak less in a way. Because I think You know when they are in pain its not like they want to listen to another lecture or another advice or things like that and I guess I'd try to listen a little bit more because they are in pain and they have thoughts and feelings that are bothering and troubling them so I'm pretty sure that they want to get that out so they don't have to suffer with those though by themselves. So, I'd listen more and if they need anything specifically rather than you know going and trying to directly medal in their business. If they want some specific favours, specific help then I'll see if I can do that and if I can then get that done as well.

I. In general, are there any factors that facilitate or help you to be compassionate towards others? When I say general I mean any circumstances or other influences in your life that have sort of taught you or helped you to be more compassionate?

P. Umm I guess you know the culture in itself helps (unclear). Umm our culture is a very umm communal community based culture where people tend to you know look into other

peoples' worries and difficulties and stick to like the family is a more stronger unit and even the extended families and friends and relations and everyone are much more closely tight together. So in a situation like that you see how different people interact and how different people you know like carry out their own version of you know values like you know sympathy understanding and things like that. So when you see that you kind of learn from that as well. And I guess you know when you have people in your life you see you know help each other out and even when I need someone's help right? And how they would help me out and from that I would learn how to reciprocate that and I guess religion also plays a role because religion kind of teaches you certain values saying you know this is how you are supposed to you know if you have a friend in a dire situation you are not supposed to turn your backs on them you are supposed to support them. Cause if you do it is pretty much one of the highest in terms of the good things you can do like the good deeds you can do in religion. And its not just that they say that you are supposed to do those things. They say those are the right things those are the things that are rewarded. So in itself it kinda teaches you how to go about doing…. the value of umm being compassionate and the value of being helpful to others.

I. yeah, okay, wonderful. Do you see any barriers that make it difficult to be compassionate towards others?

P. Um there is I guess.. Because I'm not sure how exactly to explain this one. So..one thing like it's easier to sympathise umm you know show sympathy towards a person you know closely and well. That personal relationship you have with them also plays a role. Because when a person is closer to you, you understand what they are going through, their strengths and weaknesses. Because of that, sympathising towards a person who's closer to you is somewhat easier than trying to sympathise towards a person that you don't know that well. When you're trying to sympathise towards a person that you don't really know that well, it's a bit difficult and there's social barriers as well. Because it's okay to approach a person you know closely because you know you you kind of share an amount of personal space. But if you try to help a stranger, then there is a matter of invading their privacy and things like that. And sometimes you don't know what you're getting into so because of that you know and there are always things like financial difficulties and social class and that kind of things that come into play.

I. Great! So you've told me about how you are compassionate towards other people. Now we're going to talk about how others show compassion to you.

P. Okay

I. I am now going to ask you to think about another time – when **<u>you</u>** were going through a difficult situation (or two). this could be when you were sick, failed an exam, lost a loved one or anything that upset you

P. okay

I. Can you tell me if anyone showed compassion towards you?

P. Yes definitely. Actually several, not just one

I. Okay. Why do you think that was?

P. Because I think they care right? Otherwise would they really bother you know worrying about what I'm going through or offer their help in the first place. I think they genuinely care and they are genuinely concerned about my wellbeing. So I guess that connection is what leads them to help

I. Mhh. So you think other people were being compassionate to you because they care about you.

P. That can be the case or maybe they are nice people in general and they are people who want to help

I. Okay

P. So when they saw me in a situation where I actually did need help they figured that they could you know provide help and be you know (unclear) to me so I can rise up

I. Okay I Can you tell me the things that they did or said to you that made you feel like they were being compassionate towards you?

P. So… you know the main thing is they worry and they they show that they are worried and they try to speak to me. They ask if I'm going through something and if there's anything that they can do. And there were times even if I didn't ask for help, they would go and do things to try and help me out and even if I'm not speaking to them, they would find a way to make sure I was okay. They would find someone like a loved one or someone like that and ask them. Right. Is he am I going you know am I doing okay? Am I going through a lot of problems or a lot of pressure? Is there anyway you know they can support me and things like that. So I guess you know it's a matter of not just going and doing things for me to make my life better. Sometimes it's just they show that they are worried, they show that they are concerned, and then they want to listen to my problems. , so that in itself sometimes you know when you know that there is someone who is willing to listen to you that wants to know what you are going through, and that they do care in fact that I'm going through a problematic period, or some sort of a problem, that in itself is a help.

I. What were your feelings and thoughts towards them then when they were being compassionate to you?

P. Basically I felt very highly of them. Because I actually felt fortunate that I have people like that in my life. I'm not so certain that everyone does, just … just because I do have people like that made me special in a way. And there are people who care enough to come during my difficult time and try to figure out what's going on and to offer their help in anyway possible, I guess that's a very good thing

I. Okay. It feels good to have someone around you isn't it?

P. Yes, Of course

I. And then afterwards…. How did their words and actions affect you?

P. Umm It.. it just I guess it is situational. There were sometimes, where their help and even the words of advise and their concern didn't seem like that it mattered much because maybe my situation was that dire or maybe my situ the fact that they didn't really understand my situation or at least my perspective into my situation. but then there were other times it actually genuinely felt like it mattered and their help made me feel better. So it's not just that I'm grateful for having them, I know that my life is made better because I have them in my life, that kind of people who would actually talk to me you know and. There were situations where I was given certain advice that actually directly did help.

I. Okay

P. So that means at the end of the day I knew that if I did not have them in my life I would not have thought of this solution and my situation would have been much worse.

I. Yeah. Okay.. How about them, how did it make them feel?

P. I.. I guess they were happy for me when they saw that I was doing better after a while. That like you know because when that kind of problems happen also I personally know that I tend to distance myself. So you know I'm pretty sure that also worries them. When they know that I'm okay now when I try to reconnect with them, I'm pretty sure that they feel better about it as well.

I. Would you say there were any consequences for them by helping you or ..sorry.. being compassionate to you?

P. I.. I'm not sure if they had it in mind when they did help.. but I think they might have because you know like just as I had them when I needed them, if I'm also there when they need me, I guess the fact that they helped me would you know eventually be of use for them. Or just having me as a friend I guess in the long run they might like that fact better. And I'm pretty sure they also feel good about helping me. So I guess. yeah

I. You said that you think they would also feel good about helping you. Umm.. So would you say If the same thing happened again, that you would want them to do and say the same things?

P. Of course I would want them to. Umm yeah and I would mostly want them to be you know helpful be themselves and try to support me the way they can. I'm pretty sure some of the situations would deem their responses to be a little bit different. Because you can't say the same thing in every sort of a problem. But I think I still would want them to give me words of encouragement and voice their concern and worry that would be very great.. benefit.

I. Are there any factors that facilitate or help others to be compassionate towards you?

P. Mmm.. I.. I guess you know their upbringing matters. What kind of you know what their parents have taught them what their school has taught them what society has taught them. Even what religion has taught them. And their own personal experience also I think would make them more compassionate. Because if they were in situations where they were offered help and you know their lives significantly became umm.. improved because of that, then they would know the value of being compassionate. I think they also learn that value through situations like that. so I guess experience and their learning upbringing what they have learned through different people who come in to play.

I. Okay. Umm.. This is just an extra question. But could you just explain me little bit about how your culture and upbringing and religion umm generally teaches you to be more compassionate?

P. Culture says that for our society to survive, to come to a better place, we need to be there for each other. Our culture is a communal one where people tend to look into other peoples' worries and difficulties, and stick to like family. And even the extended families and friends and relations and everyone are closely tight together. If we are divided and if we try to only serve for our own betterment and ignore the rest, that means the society in itself would crumble little by little and that would in itself.. but rather if I help others and others help me, we can actually work together and you know and we can move forward with much ease and you know with much prosperity. So even religion would pretty much say the same thing you know. Don't think about yourself. Right because you know.. you can take care of your there'll be others in a higher beam that would take care of you. so you should be more concerned about taking care of other people, cause that's the right thing you can do. So if I help other people, I'm not just helping myself. Because if I do help other people I'm doing a good thing that would benefit me as well as the other person. So I guess you know it's an act that serves multiple parties you know that would have a benefit on multiple people. So I guess that kind of encouragement is coming from religion and society, and even you know parents and loved ones. They are the kind of people who would say like here if there is a person who wants your help or who needs your help, don't fail to you know umm help them in anyway you can, doesn't mean that you have to be useful always.. but.. yeah

I. Okay. Are there any barriers again that make it difficult for others to be compassionate to you?

P. I guess yeah again you know there are social barriers. Sometimes its.. a barrier can be the fact that you don't know how to help them. because there are some situations like you know that you possess the skills or the knowledge you need to have to help them. But if you don't know.. then it's like you know you're also confused in a situation you see this person with a problem but then you're like wondering how do I help this person? I don't know. So that knowledge also comes into play. I guess experience and knowledge. As well as social factors and financial factors and things like that also come into play.

I. We are on to the third aspect now, which is about how we are compassionate towards ourselves. Do you need a break?

P. No

I. Okay. This time I am going to ask you to continue thinking about occasions when **<u>you</u>** were going through a difficult situation.

I. Do you think that you were compassionate towards yourself?

P. I am not sure.. But I would like to think that I was.. I have been.

I. Okay. Why?

P. Because you know there were situations that I was feeling down and I was not doing well and I thought about what I needed to do in order to you know get myself lift myself up from that situation to get myself to a better place. And even if that was not the case, generally I would think about my health, I would think about you know what kind of things I need to eat in order to be healthy, what kind of behaviours, what kind of activities I need to do like walking, exercising that I need to do in order to keep myself healthy. So I guess taking care of myself in a way is also being compassionate to myself. Because otherwise I can ignore about my health and wellbeing and just you know disregard all of that and go around doing whatever I feel like you know things just make me happy in a very temporary basis. But since I understand you know in order for myself to be better, be healthy and things like that, there are certain things I need to do. And… you know if I you know do exercise and if I eat right, things like that I would be healthy and much better and I'd be able to take care of myself better. So I think.. yeah.

I. Okay.. Umm Now when I asked if you were being compassionate towards yourself, you were a bit unsure.

P. Yeah

I. So, may I ask why you were unsure or are there any events that you can think of maybe where you weren't that compassionate towards yourself?

P. Because ok yeah I think so, there.. there were some situations when something like very significantly happened there were obviously things I could have done that would have made the situation better. Or you know or.. didn't make the situation worse. But.. partly I did not see the those options at that time like you know like you know like the pain and suffering I was going through and I guess umm my reactions to those did not really help me at all. So like if I was in a bad situation if I had thought you know ok I'm in a bad situation what can I do to make this situation better or get myself out of this situation, I would have been more compassionate about myself and umm you know I would have done something to improve the situation. But rather, I just saw the pain and I just wanted to inflict more pain on myself and the others around me. So I guess that in itself was not so self-compassionate.

I. Okay. How did you inflict more pain to yourself and others around you?

P. Umm I guess in my umm situation, I didn't do any physical things to cause me pain, but then I would do emotional things like you know distanting.. distancing myself from others,

isolating myself and you know if it was academics and things like that I would ignore those I would you know, if there are exams or things coming up I would just not do any work or you know no preparations or anything like that because I know that those are the things I need to do, at least to make sure that my situation doesn't become worse, but I would knowingly ignore those and you know make my situation even worse.

I. Umm.. What were your thoughts and feelings towards yourself? Umm did you feel supportive and kind or did you feel judgmental or umm what kind of thoughts?

P. Umm.. I.. It it was a mixed bag of emotions honestly. There were I guess a few like not many like like very a couple of instances maybe that I understood that my situation was bad and I indeed did things to help myself so there were few situations that I was able to show some umm self-compassion in a dire situation but most of the time I would be harsh on myself, very judgmental very critical thinking like you know how could I let you know let this happen to me and things like that and you know if I was feeling guilty I would do more things to kind of make my like I would try to wallow in it rather than trying to resonate myself from it. I would like push myself further down so I can kind of wallow in the pain wallow in the umm.. sadness and the sorrow that I'm going through rather than trying to help myself and get myself back up. So in like very dire situations I don't think that most of the time that I was able to show too much of self-compassion but rather like ignore that to a great extent and try to at least go the other way or just ignore self-compassion and just wallow in the situation that I was in.

I. Ok.. umm.. now that you spoke a little bit about the not so supportive ways of treating yourself. May I also ask was it different to the way you treated somebody else when they were going through a similar situation?

P. Very much. Because I would never try to you know push down another person when they are like you know when they are down or when they are going through a very difficult situation. I guess helping another person comes easier to me than helping myself because you feel a lot of sympathy when you see something bad happening to someone else. That same level of sympathy is very difficult to have towards yourself when you are in a difficult situation. You tend to be more critical and feel a lot of guilt. Your personal judgements about the way you acted in that situation and the guilt you have and the part that you have landed yourself in prevents you from sympathising towards yourself. That makes it difficult for you to help yourself because you are not putting yourself in the victim shoes. So, you are seeing yourself responsible, you don't really feel like you deserve the help. It is difficult to feel bad about yourself and try to help yourself'

I. Ok now coming back to the time when you were going through something difficult, how did you treat yourself?

P. Umm.. I guess there were a very few instances that I tried to support myself so I could improve, but there were a lot of situations where I was more self-destructive rather than being self-compassionate. I was I was not really trying to think of you know how I can make my situation better, but rather I would.. Not that I wanted to just okay the situation is this bad so let me just make it worse, but you know it's the pain and you know the hurt that you're going

through wouldn’t really let you do anything good to help yourself, but rather would just make you do things that would you know that in avertedly make things worse for you.

I. Umm so did you do anything umm to I don’t know to support you with the situation or to rather treat yourself quite unkindly. I don’t know anything you can think of?

P. Yeah so I guess I have done like things that would like you know there were a several instances where I have done things to kind of (unclear) my situation, but there were a lot more situations where I kinda have done things that have either hurt myself more or either hurt someone else around me more, so when they distance themselves from me that would hurt me more in return. So I guess it was I technically have done both but I think I have done the negative side a bit more than the positives.

I. Ok talking about both positive and negative things. Do you feel comfortable sharing a few things that actually did or say to do to I don’t know treat yourself?

P. Umm.. Sure. Okay so, some of the good things I’ve done is you know I I’ve figured out that the situation I was in was not good, so I reached out you know to get help from friends..umm you know to get help from.. Once there was a situation that I actually went and got help from a priest. So I had like a moral umm struggle that I was going through. I didn’t know what was the thing that I was supposed to do in that situation, so I was struggling with it so went and spoke to a priest and got help you know and found what I needed to do, and that kind of did help. But on the flip side then there were situations where I could have supported myself more, I tried distancing myself from others, you know isolating myself, or getting into negative habits, right? So.. I guess I have done a lot of that so.. yeah.

I. Did you do anything positive to look after yourself, that you can think of?

P. Umm.. Yes I have you know there were times you know like even when I had illnesses and things like that I would know that I am going through a certain thing so you know I would take vitamins and I would go and meet a doctor, get a prescription, get the medication and take care of myself. Because that would technically count as a situation that I needed to be positive towards myself. And I actually did go that extra mile and got the help I needed and yeah took care of myself.

I. Were there any consequences again for you from treating yourself the way you did?

P. I don’t think there was anything negative about it. If I if I did help myself (unclear) the outcome was mostly positive. So you know if it was illness or something like that I recovered much faster and in the situation of that moral dilemma I was going that moral umm situation also the advice that priest offered did help to a certain extent, it did not completely resolve the issue, but with that advice and you know with some more things that I could gather myself, I was able to figure out where I needed to go from that and I kind of solved the problem, so it did help.

I. Okay. If the same thing happened again, would you do and think the same things about yourself?

P. I yeah I think I guess you know trying to be a more little bit more compassionate indeed would benefit me rather than trying to be self-destructive. Because I really can't think of a situation where my self-destructive practices benefited me in anyway. But self umm self-compassion and being understanding as to what I need to make myself or get myself into a better situation actually has helped. So I would actually try to be a little bit more positive and little bit more compassionate towards myself.

I. What things would you do to be more compassionate towards yourself?

P. If it's something that I know how to like improve, how to make it better, I would try to immediately go and you know get around to that. But if it's something that I don't know how to resolve by myself, I would reach out.. I would talk to a person whether it is a friend or whether it is a loved one or whether it is something umm labels (unclear) I would talk to a professional, whether it is a priest whether it is a psychiatrist or whatever it is you know the help that I can get I would speak to that person and I would try to get that help and resolve my issue.

I. Are there any factors that facilitate or help you to be compassionate towards yourself?

P. Umm.. I guess. The amount of sympathy you can show to towards yourself matters. And.. and.. the amount of understanding that you have about your emotions and how you process them you know because if you are angry how you go about doing you know what you do, and if you are sad what do you usually go around doing, so if you understand what you normally do when a crisis hits you.. that makes it easier to understand what you go through and kind of helps you to figure out what you need to do from there.

I. Okay.. I guess my question is are there anything in your life that have shaped you to be more self-compassionate? Like your upbringing or you know teachings and anything of that sort?

P. Yeah.. so upbringing helps a lot. You know you learn that you know its not just the fact of taking care of others. You need to be able to take care of yourself. I see how my parents take care of themselves, right? Like when they are sick they don't just ignore that, they go to a doctor and get the medication that they need, right? And even in religion it says that you are not just supposed to take care of other people you need to take care of yourself. so those kinds of values show that it's not enough that you try to be generous and helpful towards other people, you need to be helpful and generous towards yourself, So that you can be that then you are available to be that when someone needs that as well because if you don't take care of yourself you're not going to be in a position to help someone else when they are in need. I guess upbringing even society I guess matters a lot in this. ..(unclear).. Its kind of how people co-exist helping each other and how they help themselves as well, both of them matters in order for people to kind of you know grow as a society grow as a society and evolve.

I. We are on to the final question now. Are there any barriers that make it difficult to be compassionate towards yourself?

P. Umm you know I guess one of the things is the fact that you don't know how umm you release or process your emotions. Emotional understanding you know. You wouldn't be able to help yourself if you can't see the situation you are in. Right? and you know those emotions are kind of the indicator that says ok you are feeling sad, you are feeling distressed or you are not in a good situation.. so.. your ability to see your emotions, your ability to kind of understand what kind of emotions you are having kind of helps you to figure out your situation and you know be more open to yourself.. be more helpful to yourself as well. And the same time I guess again the matter of you know knowledge comes in you know even to help yourself you need to know how to understand a situation and how you need to proceed with it and the other people you have because sometimes helping yourself means you need to go and get help from someone else so you know the society people around you matters. Again things like financial boundaries, things like that.. those also matter. And then just you know matter of when you need help sometimes you need to have to even to know that you need help you need other people to kind of pointing it out that you are not doing so well and you need help so you know having good support from society whether it maybe your parents whether its your friends or other people in society, you need to have that. So if you don't have that kind of a good society to back you up when you need help, then that would make it more difficult to be more self-compassionate towards yourself as well.

I. Would you say your society is more supportive or not so supportive?

P. I think my society is very supportive. I think they are kind of built in that culture where you are not supposed to turn ….around when… there's someone who needs help, but rather try to approach them with what they need and help in the best way you can. So kind of society not only helps you when you need help the society kind of teaches you as well. When someone needs help that you should help them and when you need help there are people around that would in fact help you as well.

I. Okay then. That's the end of the interview. Thank you very much for your participation. Do you have any questions?

P. Thank you. Not really.

Interview 2.

I. Ok. So the first question, can you tell me what the term compassion means to you?

P. Umm

I. So it doesn't have to be an exact definition, anything that comes to your mind when you hear the term compassion.

P. Empathy, like being I think empathy goes hand in hand with compassion and that the ability to feel sympathy makes us compassionate towards something. Or.. or even like being empathetic towards yourself also.. like basically I think both go hand in hand.

I. Can you think of any other other words other than empathy that reminds you of compassion?

P. Umm.. yeah.. consideration towards feelings and other peoples' needs and our own needs.

I. Ok.. So the second question can you talk about your understanding of self-compassion?

P. My understanding is it is a process. Umm… you know.. It is basically a goal that needs to be reached through a process where you are able to show love and care towards yourself. But it doesn't happen overnight. And you need to work towards it and be aware of what your needs are and who you are to be compassionate towards yourself.

I. So the next set of questions are going to focus on three aspects of compassion. The first one is how we show compassion to other people, the second is how others show compassion to us, and the third is how we are compassionate towards ourselves.

I. So for the first one…I'd like you to think about one or two occasions when a loved one was going through a tough time or difficult situation. This could be a family member or close friend and could be an event where they were sick, failed an exam, lost a loved one or anything that upset them. Okay?

P. Okay. You want me to tell you the situation?

I. Umm no you don't have to tell me the situation, but I want you to recall some event in your mind. Have you thought of any situation like that?

P. Yes

I. Now can you tell me if you showed compassion towards them?

P. Umm I think so. In my in my own way of doing it, yes I think so. I mean it might not have been perfect, but yeah.

I. Okay, you tried to be compassionate towards them.

I. Ok. Can you tell me the things that you did or said to them?

P. Umm first thing is that I made sure that they didn't feel judged or that they didn't feel like they were being scrutinised for something that they did. Umm that's something I did, so. And something I said would be something like you are only human, people do people make mistakes and you have the space to make everyone has the space to make mistakes and so do you. And then sometimes you need to give yourself the opportunity to learn from it, and yeah.

I. What were your feelings and thoughts towards them? How did you feel about them?

P. Umm.. how I felt about them was..

I. Yeah.. About them and the specific situation that they were going through.

P. So initial feeling was sympathy because I felt bad for them but because they were feeling guilty about something they did, but umm but moving on from sympathy.. I just wanted to make sure that they had somebody to talk to when they umm while they were going through that. So I didn't want them to feel alone and then I wanted to make them feel like this like can happen to anybody and that you know it's not something it's not the end of the world and it's it's fine and it's okay to okay to have done whatever they did.

I. Okay, umm and then afterwards…. How did your words and actions affect them?

P. Umm.. I hope they felt better (laughs) but I also I also think they felt that they were not alone and that they could like (unclear) actually like think about it in in a way that they see the bigger picture think that I think I helped with the problem, at least a little bit at least like for a temporary finding.

I. Okay, so how about you? Was there an impact on you?

P. (Clears throat). Yeah of course. I mean I like it's when when somebody is talking about their problems and, most of the time you can always relate to it, their problems or mistakes you can most of the time relate to it. So yeah I thought about how at that time I thought about how I dealt with the situation and basically I told that person not to be not to be so harsh on yourself as I was with myself, so I learned from my mistake and was able to help or tell them not to beat themselves when they make mistakes or when they feel guilty about something, I was able to relate to it.

I. Okay. Did it have any negative impact on you emotionally or or in any other way?

P. Umm yeah because this person was my friend and I felt bad. And I also felt kind of after talking to them I also felt kind of responsible. I felt responsible if they felt worse about the situation afterwards because you question yourself, right? If you might have said the wrong thing or sounded judgemental.

I. Okay..If the same thing happened again, would you do and say the same things?

P. Umm.. I wouldn't say the same thing, but I definitely again wouldn't be judgmental about it because I mean there's no point judging people right, I mean people I mean some people are able to learn quickly from the mistakes and some people are not and. So.. if she or he did whatever he or she felt was right at the time or if it was a irrational quick spontaneous decision and they did that..I mean it happens, so.. other than being there for them you can't

really tell them anything differently. I..I would still try and be empathetic towards it rather than be harsh and say oh why did you do it again. I wouldn’t do that.

I. Are there any factors that facilitate or help you to be compassionate towards others? So.. are there any things that you learned in your life or people who influenced you to be more compassionate or any other factors that you think have made you a compassionate person towards others.

P. Umm definitely yes.. Umm.. I guess like just experiences and listening to people or reading things. And seeing how how as much as as unique as we are we also most of the time share share the common problems and common issues. So.. so that being aware of your surroundings and the people around you definitely and listening to them definitely makes you compassionate. And (laughs) as stupid as this sounds also animals make you compassionate because their the unconditional love they show you kind of makes you question everything and question your ability to dedicate yourself to something. So, definitely animals as well.

I. Since you spoke about the surroundings and therefore animals as well, would you say there have been any cultural or religious influences as well, or not?

P. Umm.. in general or for me?

I. For you.. for you towards becoming a compassionate person to other people.

P. Having having being religious before.. has actually taught me how how I can be compassionate even without religion or without the teachings of a religion. And.. and culture of course I mean definitely influences people and has influenced me as well. Because.. like for example If I talk about the culture that I am living in, people are compassionate, but if you start comparing this culture and another culture, it’s definitely different. They are only compassionate towards people who share their same beliefs and who are in their same belief system. But when that changes a little bit, you are either from a different religion, or different racial background or different educational or socio-economical background, that compassion changes. So obviously this compassion is very conditional towards the person’s background. That’s how I see culture has influenced this society. Therefore, having been religious before, has actually taught me how I can be compassionate even without religion or the teachings of a religion. But for me I am able to see it as like everybody, I think compassion also comes with acceptance of who people are like that everyone is different and that’s okay. So culture has definitely affected me more like in the sense that it has taught me like sometimes it has taught me how not to be. You know.. and same with religion. That you don’t have to be cultural or religious to be compassionate towards something.

I. Okay.. same question but a different direction. Are there any barriers that make it difficult to be compassionate towards others?

P. Umm.. Yes. Something like for example like if when people commit or when people do things that are really really hard for you to accept like for example being cruel to animals or being mean to kids or being being judged for being a woman for wearing something the society thinks is indecent or uncultured and those things are very like when people are mean to you, it's hard to be compassionate towards them. You know, because like why should I be compassionate to you when you're being so mean to me.. but yeah. I think personal factors like factors that daily affect you, if it affects you in a negative way I think that affects your compassion towards the person who's showing that emotion to you. I don't know if I make sense.

I. Of course you do. So you've told me about how you are compassionate towards other people. Now we're going to talk about how others show compassion to you. I am now going to ask you to think about another time – when you were going through a difficult situation or two. e.g., this could be when you were sick, failed an exam, lost a loved one or anything that upset you. So previously we spoke about when someone else was going through a tough time, now I want you to think about a time when you were going through a tough time.

P. Ah okay.

I. Can you tell me if anyone showed compassion towards you?

P. Yeah definitely, I think so I mean a lot of people did a lot of close friends did.

I. Why do you think that was?

P. Why do I think they showed me compassion? Umm one thing because I think they all they really loved me and they really cared and worried about me or worried about what had happened and they wanted to be there for me and didn't want me to feel alone.

I. Can you tell me the things that they did or said to you?

P. Mmm.. they said umm they said things like with time it will be fine and umm not to be too hard on myself and that you know it's all just temporary and it will be fine.. things like that.. yeah.

I. So when they were being compassionate.. what were your feelings and thoughts towards them?

P. Umm.. at that.. (Laughs). At the moment obviously I'd be like oh no.. everything will go wrong and no nothing will be fine but then obviously that umm more than about what they said the fact that they were there to help me and just that their presence helped me in knowing that okay I'm I'm not doing this alone and there are people who are there who are there for me when I do fall down. So it's just their presence and when when you are in when you're

having a problems and when people tell you things like you don't really accept it at that moment right.. but later when I think about it afterwards it made sense it it helped a lot.

I. I know you answered the next question but I will ask it again. And then afterwards…. How did their words and actions affect you? I think you spoke about it but..

P. Umm.. yeah it helped a lot and it kinda makes you reflect on like again I keep saying this like the fact that you feel like you are not alone and that this thing happens to everybody and the fact that you can relate to somebody else and the confidence that you get that okay they got through it so I can get through it as well. So that feeling really helps.

I. How about them - How did it make them feel? Or was there an impact for them?

P. Umm.. I.. I think they might have still been worried a little but I'm sure they also felt better about the fact that they were able to be there for me and that they were able to help me out. And.. yeah and and to have gone through the progress of me recovering from that. I think yeah that would have definitely I mean obviously they helped right. That's why somebody has recovered from it. So I think they also would have felt good about it.

I. If the same thing happened again, would you want them to do and say the same things?

P. Yeah definitely.. Yeah.

I. Why

P. I mean ob obviously there have been people who have not been compassionate as well. But when they were compassionate, yes I would want them to say and do the same things.

I. Why? Why would you want them to do and say the same things?

P. Because because genuinely it did help. Because again I I I felt like they could relate to me and they made sense in a way that like I've been through this too so.. it's fine that you're going through this now. You will also get through this like you will also overcome this. It's just that confidence you get.. yeah.

I. Are there any factors that facilitate or help others to be compassionate towards you?

P. Umm.. yes. Definitely my attitude and my relationship with this person. But if you are talking about strangers being compassionate, then I guess just I don't know I'm sure it's a lot of external factors like their mood, their day and.. but then what what about me that made them compassionate? I'm not sure maybe they felt bad. Maybe they they felt bad they how they maybe they could remember how they felt at the time and they wanted to help somebody. So.. I don't know maybe something like that.

I. Are there any barriers that make it difficult for others to be compassionate towards you?

P. Any barriers for others?

I. Mmm hhh..

P. Umm.. Yeah I guess every day's factors. Like mood and.. your daily life how like your situations like how your day went on the day.. your attitude towards the person.. yeah.

I. We are on to the last aspect now, which is about how we are compassionate towards ourselves. Umm.. again this time I am going to ask you to continue thinking about occasions when you were going through a difficult time. Okay?

P. Okay

I. Umm.. Do you think that you were compassionate towards yourself? Umm.. we

P. No

I. Sorry.. Sorry for interrupting. We spoke about how you showed compassion to other people and seemed like you were happy to receive compassion from others. So when you were in this situation were you being compassionate towards yourself?

P. Umm.. I don't think so. Now that I look back and think of it.. I definitely wasn't. Umm.. I umm.. could have definitely been more compassionate towards myself. But.. I wasn't. I don't think I was.

I. Why do you think you weren't?

P. Umm I think it's because I kind of tend to blame yourself a lot for something that goes wrong even if that thing is beyond your control. You still try and put blame on something right? So you tend to blame yourself. And I blamed blamed myself a lot and in that process I lost my compassion for myself and then I for forgot to just give myself some space to make mistakes.. yeah.

I. What were your thoughts and feelings towards yourself?

P. (Clears throat). Umm.. things like I don't know like why why would I do it or why would I keep doing it e even when I know it's wrong or when I know it's not working.. and.. like it's just constant blame and constant feeling of like you know you're not good enough or you're not it's only things go wrong because you're not good at it or you're not good enough or you're not great enough to do this or to pull this off. So.. I think that's why.

I. So I asked about the thoughts and feelings you had. How did you treat yourself?

P. (Laughs). Umm.. Well treat how did I treat myself? I think it's just that not giving myself enough space to breathe and enough space to just relax a little bit.. and then like I didn't take care of myself like health wise. I just let go and I just completely went you know just completely went overboard.. didn't exercise.. didn't drink enough water didn't eat.. didn't sleep properly. Didn't care enough to sleep properly.. and that affected like my physical and mental health a lot not getting enough sleep. So…yeah.

I. Again you answered my next question a little bit as well. Were there any consequences for you from treating yourself the way you did? Besides not having enough sleep.

P. Were there any other consequences of not getting enough sleep or?

I. Not not getting enough sleep but were there any other consequences for you from the way you treated yourself? With lack of compassion?

P. Yeah definitely. There was like again it's like a spiral right? It's like like a cycle of like you you treat yourself without compassion and then you go back to being worse and then again you treat yourself so it's a cycle of blame and it keeps happening again and again until you come to the realisation that you need to get your acts together. But definitely consequences like you know you.. you.. you're not able to concentrate properly you're not able to ta talk to other people properly. You're not able to maintain.. it affects your relationship with other people. It affects your relationship with like other things like your education, your social life, you're your hobbies, things you like. You know just your daily routine gets disrupted because you're not being because I was not being compassionate with myself.

I. If the same thing happened again, would you do and think the same things about yourself?

P. Umm.. there's definitely a chance that that might happen because we are all human and I think it tends to happen that we blame ourselves more than we should. But, I think I have learned enough and have had enough reflection time.. self reflection time to not make that same mistake again and try and get myself a little I I might I'm sure I'll be a little bit better than the last time.

I. Okay. Can I ask an extra question? You were showing compassion to other people and you were okay with receiving compassion from other people. Why do you think there is a difference between that and treating yourself differently?

P. Umm (laughs) that's a good question. (laughs again).. But I think we always put ourselves in a box where we are so protected and guarded but then we want each and every one of our efforts to be impressed or acknowledged or recognised. We do not give that space to

ourselves as much as we do for others. I think we are harsh on ourselves and judge ourselves harder than we judge others. Yeah that's why.

I. That makes sense. Okay.. Are there any factors that facilitate or help you to be compassionate towards yourself?

P. Yes.. Umm external factors. Umm… umm.. I would say external factors would be like.. okay more than external factors I'll just talk about like self-reflections and thinking about your actions and the consequences. Thinking about it in a not in a judgemental way but like in a reflective way.. like okay I did this.. I did this.. is it okay that I did this? So.. it's like… and also learning from others. So.. and that would be an external. Just to see how other people get through their difficult times and how they are compassionate towards themselves.. and that also helps. Yeah.

I. Okay. The last question.. are there any barriers that make it difficult to be compassionate towards yourself?

P. Umm.. yes.. Again.. your expectancy like how much you expect of the situation and if it doesn't go well, then you're hard on yourself. And then.. umm.. also things like.. like again external factors like.. when others are mean or judgemental to you it's difficult for you to be compassionate towards yourself. You take other peoples' views into account of how you should treat yourself. When somebody is mean to you when you're sad, you start thinking ok maybe it's my fault, maybe what I did was so wrong that I cannot forgive myself.

I. Umm.. Would there be any social norms or anything like that, that would also negatively impact on becoming compassionate? Or societal cultural influences..

P. Yes.. social norms like.. yeah.. umm.. definitely norms like.. umm.. you know the the.. stereotypic view towards women.. I think.. like if if if I go out for example, if I go out at night and I get assaulted.. it's because the mistake is mine because I went out at night because girls are not supposed to go out at night. But, if the same thing happened to a boy, it's probably the fault of whoever committed the assault. So.. things like that societal, stereotypes and stigma towards women towards religion, race, culture, language everything I think is is a problem that affects peoples' compassion towards others.

I. Okay that's the end of the interview. Do you have any questions?

P. No..

Interview 3.

I. So for the first question…. Can you tell me what the term compassion means to you?

P. Umm.. term compassion is umm.. in terms of what I understand is like being really loving, kind and empathetic towards myself and towards others.

I. Okay.. Speak a little bit loudly.. Umm.. you don't have to know any exact definition, that's exactly what I expected. So for the second question can you talk about your understanding of self-compassion?

P. Mmm.. self-compassion is as I told you.. Like we have to be very loving and kind towards ourselves. And.. it's I think loving others come after umm loving yourself. First you you need to understand who you are and what kind of person you are, and how important you are and you should be able to respect yourself and love yourself and accept the good qualities and as well as the flaws in you and not judge yourself umm and.. just be.. very compassionate towards yourself and with that training you are able to be compassionate towards someone else. If.. if you are not compassionate towards yourself I think that person would not know how to be compassionate towards someone else.

I. The next set of questions are going to focus on three aspects of compassion. The first one is how we show compassion to other people, second is how others show compassion to us, and the third is how we are compassionate towards ourselves. So for the first one… I'd like you to think about one or two occasions when a loved one was going through a tough time or difficult situation. this could be a family member or close friend

I. Can you tell me if you showed compassion towards them?

P. Yes.. Umm actually umm at a time like that you need to be umm non judgmental and try to understand their problem, try to umm I mean go in to their shoes and try to understand as if it has happened to me and then think like what will I do if I'm the person who's going through this right now. Anyways, like I am not judgmental at all towards others as well as myself, so I have tried to help them in many ways like tried to be ummm you wanted to know how I helped them right? So.. tried to make them understand actually tried to make them understand by themselves about the situation and the big picture umm of the situation.. and it had been successful umm many times.

I. Could you tell me why you decided to show compassion to them?

P. Umm because they came to me for like for help and then because they came to me for help, I wanted to help them the most I mean the best way I could…so that's why I usually when I'm helping someone in a difficult situation, I try to be non judgmental and listen to them properly and be empathetic and compassionate towards them.

I. You already told me that you try to be empathetic and compassionate.. but can you tell me the things that you did or said to them? Can you tell me in particular, these are the things I told them and these are the things I did?

P. Ok.. So.. Umm actually I.. um I just asked their story and then I just umm want I just asked them to umm discuss the complete scenario in different aspects. Umm.. so they also can understand sometimes they just umm.. they just look at the problem in one point of view umm..and there are other other pathways that they don't see at that time, so I just try to I asked them questions umm… where they can explain and they themselves can understand umm kind of what is wrong with this and what is wrong with them umm.. and why are they suffering like that at that moment and then through that most of the time they see other pathways that they can they can umm come out of their problem.

I. Ok. What were your thoughts and feelings towards them?

P. Umm.. at that moment?

I. Yes. When someone you love was going through something?

P. Umm.. I tried to be um.. like very kind of um.. kind of kind and sometimes I see that it's it's not a big deal that they have made the situation like worse by themselves. And it it's not anything to like umm.. anything to be that much worse but then at the same time I don't judge them..

Interrupted by an outsider.

P. Umm.. so I.. but sometimes umm.. just sometimes I see that it's not something umm.. which is not a big deal but then I still try not to judge them and think it's silly or something like that because according to them um.. in their perspective because they don't see the actual picture of the problem.. they go through that suffering.. so I feel sorry for them and I try to be very kind and loving because otherwise if you don't if you don't show them loving and loving kindness they will not be able to I won't be able to help them, because they will just go away from me.

I. And then afterwards…. How did your words and actions affect them?

P. Umm.. most of the time I see um.. that they listen to me and they try to understand. And umm.. lot of people have told me that they feel really good after talking to me. Sometimes it's actually being a little distancing as well because they keep coming back to me and with my work I feel distracting. To tell the truth, I have been irritated.

I. It's interesting that you said it's distracting you because my next question is how about you? Was there an impact on you by being compassionate towards them?

P. Umm.. yeah sometimes as I told you. But then I tell myself also that there should be a limit umm.. to things and also sometimes if I know that person is quite okay and that they are able

to handle themselves, after after ummm being compassionate and trying to help when they come out their problem, then when I see that after this they don't need me often and that they can handle themselves by themselves, but still they are like because they feel comfortable talking to me they reach me often for even the slightest thing. So, sometimes I tell myself that this should have a limit, and then sometimes I knowing that my umm.. advice and my help is not necessary, I sometimes keep a small distance from them. Otherwise it's it's not going to work for me as well as for them.

I. Has it affected you in anyway when people come back over and over again for help?

P. Umm.. sometimes to tell you the truth, sometimes in one or two cases I have been like kind of irritated. But.. but I .. I .. I don't show them that I'm irritated and I just try to quickly tell them something or just.. talk about the situation and dismiss them.

I. Okay. If the same thing happened again, would you do and say the same things, based on your experience?

P. Umm.. it's always it depends on the situation and umm.. what that person's personality and then depends on how that person is umm.. looking at the problem and… then things work for that person, which things work for that person, doesn't work for that person. So I always take.. take those things and.. just I don't give the same set of advice or things like that to the other person who has the same problem. I just try to see if this person what this person is like and the person's personality and how that person is looking at the problem. There's always differences.. no two people will look at the same problem in same angles.

I. Yeah. Okay.. Are there any factors that facilitate or help you to be compassionate towards others? So.. are there any circumstances, people, or the way you were brought up?

P. Umm.. yeah. Yes. I actually I was.. From my childhood I was kind of compassionate to people. And there were times that I was they had been using me to like.. because at that time I didn't know that it it was I'm not talking about any abuse or something. It was just it.. was they just were are like really dependent on me which was.. and umm.. expecting help from me all the time.. and I had been doing that helping them unconditionally like.. I mean umm.. without umm.. I mean sacrificing umm.. my things also.. But then after I started umm.. learning umm.. psychology and after I started working with this psychological background, then I realised that ummm..there should be limits and also I.. I have kind of like.. values. Yeah. I have values. When I'm compassionate towards others, I use them properly and also being in a very religious, we are from a family very helpful, religious and cultural background, so I think those things also mattered in who I am.

I. Are there any barriers that make it difficult to be compassionate towards others?

P. Okay.. Umm.. there are I don't think any barriers in my life.. It's.. Umm.. no. So far there are no no barriers that I can help anyone anytime.

I. Brilliant. So you've told me about how you are compassionate towards other people. Now we're going to talk about how others show compassion to you. I am now going to ask you to think about another time – when you were going through a difficult situation or two.

Can you tell me if anyone showed compassion towards you?

P. Umm.. yes actually I have umm.. few very good friends. So they umm they have compassion towards me. And.. I feel free to talk with them and discuss things. But at the same time I can also umm.. as I told you because I am studying this field, I am able to umm.. kind of analyse be aware and analyse umm.. why am I feeling this way and why what should I do next and why, why did it happen like this? Things like that I can analyse myself as well. But if I, if I need others' compassion, I really talk to them.

I. Why do you think they showed compassion to you?

P. Umm.. I think maybe because we are really good friends and we connect well with each other. And we respect each other and umm..they are also very non judgmental. I think we all talk about these things with each other, so they also understand what it means umm… to be non judgmental and being empathetic towards others.

I. Can you think of the things that they have done or said to you that made you feel like they were being compassionate to you?

P. Umm.. They like umm.. they follow up. They don't like talk to once and let you go. But, they follow up and they they keep asking me , if everything okay? Is everything working out for you the way you wanted and if there's anything else, we can still talk and umm.. and they try to cheer me up and then we try to go out for like like for a change, we do things like that.

I. What were your feelings and thoughts towards them?

P. I feel very grateful to them and I I.. feel that they are like my own family. Umm.. yeah.

I. And then afterwards…. How did their words and actions affect you? How did you feel when they helped you?

P. Yeah, obviously like always it's in a positive way. But sometimes, umm.. at times I have felt that when they when they tell me something or maybe we could meet kind of I..I have felt that this is not going to work for me, like in me. But I don't tell them you know this is not going to work, what you're saying.. I I tell them but sometimes if I have the feeling that it's not going to help, I try to figure out things by myself.

I. How about them - How did it make them feel?

P. Umm… I think they were quite satisfied and they felt umm.. they they..felt like they umm.. helped me and they.. they wanted to follow up and ask me things. So I think umm always they.. felt I think they felt good.

I. If the same thing happened again, would you want them to do and say the same things?

P. Umm.. no we always talk about things, so I just want to I just want to.. umm I just don't want to stay away from them and try to umm.. handle my own things, I just because I know that they're helpful and they are understanding and they are non-judgmental, I I.. always talk to them.

I. Are there any factors that facilitate or help others to be compassionate towards you?

P. Umm.. yeah of course. They have they also have good backgrounds like they don't … they are not in a disturbing background, they understand things. And also because we talk about things like, because we talk about people and umm.. how good bad happy sad and what should be changed and what should not be changed, things like that we discuss. So because of that amongst us we have a .. we have a understanding, that we all we all kind of have.. umm.. similar mentality.

I. Are there any barriers that make it difficult for others to be compassionate towards you?

P. Umm.. these few friends umm.. no there are no barriers. But like some some of them are married. So sometimes they have family commitments, so whenever if I need to talk right now, sometimes they won't be able to talk to me. But still they umm… they get back to me as soon as they find time, but then I understand that I understand that they also have their own commitments and, It's important for them that I should I should step back a little and umm.. give them sometime. And…Because I'm not a very restless person, I can… I can just wait for them.

I. Sometimes when people have family and other commitments, it is difficult for them to commit. We are on to the third aspect now, which is about how we are compassionate towards ourselves. This time I am going to ask you to continue thinking about occasions when you were going through a difficult situation. Do you think that you were compassionate towards yourself?

P. Umm.. yes. Umm.. Yes.. Sometimes, it's not like perfectly compassionate. Sometimes like I think that umm.. there are umm.. there are times that I become a little judgmental about my myself as well. Nothing is perfect right? but.. being.. after being judgmental then again I… I can think that it's anyway it's I… I cannot correct it now, it's all in the past.. So.. there's no point of worrying about it or going on worrying about it too much.. umm.. so.. I can actually I can be quite mindful about my thoughts because because I practice mindfulness, so it helps.

I. Why do you think you were compassionate? From what I understand would you say you were as much compassionate towards yourself as you were to others?

P. Umm yes I think it's the same. It's the same way because I'm I'm used to I'm used to kind of like a way of being compassionate and showing compassionate compassion, sorry. So looking at others, I look at myself the same way as I look at others.

I. Why do you think you show compassion to yourself?

P. I understand that umm.. if if I don't show compassion to myself, umm I can't expect it from others. Like sometimes even earlier days, it's been like I've always tried to figure out umm.. I expect something from others, but then I don't get it. And then umm.. later on I.. I thought what I'm expecting and umm.. what I'm expecting that I should give to myself. Because others are like they have different mind sets different, they are not me they're different people. So I can't exactly expect what I need from them. So.. if I need something, then I should be able to give it to myself. Like like that I try to figure things out.

I. What were your thoughts and feelings towards yourself when you were going through a tough time? How did you feel?

P. Umm.. there are times which I have felt terrible. Umm.. to tell you the truth, but then again I.. I tried to kind of analyse, why do I feel like this and what are the reasons, and then I try to understand if I can change those reasons. Then if I can't change those reasons, I try to tell myself that it's okay, now it's all happened and now I can't go back and change anything so I need to go forward from here and will just go with the flow.. and ummm try to take one step at a time, like that I can umm analyse and umm do things for myself.

I. How did you treat yourself? What are the things that you did to look after yourself?

P. Umm.. sometimes it's really stressful. Even analysing it and trying to figure out things is kind of stressful. So what I do is like I try to keep myself like umm.. entertaining with my hobbies and I can understand that if I neglect all that, I will be in a very distressful situation and that I should I should try to relax, and then I try to do things for relaxation and go to places where I like to go or watch, I like watching movies. So I try to watch movies and then read or listen to music, things like that. I don't completely umm get get indulged in that situation and get more stressed and distressed and then umm stay without doing anything. I still like to keep myself calm. I usually tell myself, sometimes I talk to myself and I tell myself that it's okay that you need to just.. umm you need to just get up and get dressed and go. Like things like that and you need to right now you need to relax. Things like that.

I. Were there any consequences for you from treating yourself the way you did? What were they? So you were going through a tough time and then you spoke to yourself, thought of what you can do, sometimes you took yourself out for movies, so how did you feel?

P. Yeah so I felt relaxed and then.. the problem is still there.. but then when I'm relaxed my my brain works better like I can concentrate more on things, and then I don't take things as much seriously as I used to.. and then then I try… I try to umm look at them in a in a.. better point of view.

I. If the same thing happened again, would you do and think the same things about yourself?

P. Umm.. no basically I think I would do the same. Because I'm used to a system that I have made up to keep myself calm and happy. So I think I'm quite comfortable in that system. So I will.. follow the same thing.

I. Are there any factors that facilitate or help you to be compassionate towards yourself? Any people, background any circumstance?

P. Umm.. Actually I learned about compassion through meditation. I've been doing this Buddhist meditation for the past 10 years. It gives you a deep understanding of the things in mind and matter. So, you're able to analyse and understand things that, it's happening because of this and what needs to be changed, and if it cannot be changed, you have to just accept it. Most of the time it's all about accepting, accepting the present moment and living in the present moment, going step by step and going with the flow. So I understand if you if you like jump out of the present moment and if you go really back, you get depressed. Back in the sense like if you go to the past you feel depression, like depressed. Because we always umm.. in the past we think like we couldn't get this done and this happened not according to my umm.. how I wanted and things like that. And also if you think too much about the future, you become like scared. It's you feel anxious and anxious and you tend to feel will this happen, what if it doesn't happen this way and it's also really disturbing. Because understanding those I try but there are times, I mean there are times obviously I go like umm.. to the past and the future, but umm.. at the same time, I am much more.. my mind is much more trained to keep myself calm and think about things in a umm.. in the present moment.

I. Umm.. Can I ask you an extra question because you said that you've been meditating for a couple of years now. What got you into meditation? Is it coming from childhood or were you more interested after a certain thing or?

P. Umm.. yes, because umm.. no it didn't come from the childhood. But our family was quite religious. So we had an understanding of all aspects of religion. But then at one point, I.. I wanted.. like I.. umm.. I initiated a liking to try. Mostly.. I like to try out things, so I wanted to try meditation. But then I didn't get a chance. So.. for a few years I had that idea in my mind but I didn't get a chance. And also.. there was a difficult period in my life with relationships. So at that time this trying meditation really came to my mind. So.. I thought okay I'm going to try this somehow. And then.. I.. Because earlier, I think I didn't quite go to meditation because I didn't have any problem. But with these relationship problems, then I wanted to try this out and how this can keep me calm and how it will help me to realise things and accept things and let go of things, like that.

I. Are there any barriers that make it difficult to be compassionate towards yourself?

P. Umm.. no. not that I know of anything like that.

Interview 4.

I. Can you tell me what the term compassion means to you?

P. Compassion to me is something that you are born with and umm.. compassion is something you can give yourself and also umm.. give others. Compassion can be in the form of love, so where there is love, there is an act of compassion for me.

I. That's quite a statement (laughs) Can you talk about your understanding of self-compassion?

P. Self-compassion? So self compassion is yeah basically it is love for yourself umm.. that you don't forget to love yourself and in order to love others or to share your love with others, you have to have self-compassion. That means love to love towards you, to yourself.

I. Brilliant, So.. the next set of questions are going to focus on three aspects of compassion. The first one is how we show compassion to other people, the second is how others show compassion to us, and the third is how we are compassionate towards ourselves.

P. okay.

I. So for the first one…I'd like you to think about one or two occasions when a loved one was going through a tough time or difficult situation, this could be a family member or close friend and could be a time when they were sick or anything that was upsetting. Can you tell me if you showed compassion towards them?

P. Umm.. yes I did because that was when umm.. one of my family members was sick and I had to show it. I mean if if, if it wasn't for me nobody else was there to show it. So, understood, I understood basically that you know.. the form of compassion like say helping them by their bed or anything anytime they wanted anything, so I had to be by their side and I had to make sure that everything they needed I mean she needed was there for her.

I. I know you explained me why you showed compassion to them. But could elaborate a little bit more as to why you were showing compassion to them?

P. Yeah ok. So one thing because she's my mother. So.. generally I have this thing I have in my mind small age that she has been doing stuff for me at any cost you know, umm being being there for me. So I can't think of anybody else who could be there when in her times of

trouble or may be sickness. So.. one thing I should be there, and number two I had to be there and number three, it’s my responsibility to be there.

I. Can you tell me the things that you did or said to them which makes you believe that you were showing compassion?

P. Ok. So she was on bed, that means she was down with the dengue fever. And before she was properly admitted, she had to take bed rest at home and she had to cut down on her housework. So I showed compassion was I brought food to her and whenever she wanted to umm.. go to toilet I helped her and held her and even with medication, whenever the doctor has subscribed umm., prescribed medicine I gave her on time and I was by her side and umm.. and ummm.. I made sure that she didn’t’ feel lonely just because she was in a room and yeah.

I. What were your feelings and thoughts towards her?

P. So.. I wished that she would soon recover and get out of bed. That is what I wanted and I too myself felt so empty and I felt like I lost something, because when she was away from bed and like how she normally is, she did a lot to me like in food, in clothing say housework. She was a big hand, so when she had to halt on a bed it was a very big difference to me and my other family members. and.. yeah

I. And then after you showed compassion to her…. How did your words and actions affect them?

P. Umm.. I really don’t know how she felt (laughs) but but whenever I spoke to her, whenever I said I want you ‘amma’ back you know out of bed again, you know I want you recovered soon, so I could see that she was happy and I’m sure she could feel how much I missed her normal being.

I. How about you? Was there any impact on you from being compassionate?

P. I feel like whenever I’m compassionate to someone, I.. I feel like this thing, umm.. I feel whenever I become compassionate to someone, I feel like this vibe going out of me. I feel good when I show love to someone, it’s a self-satisfying thing for me.

I. If the same thing happened again, let’s hope not , but would you do and say the same things?

P. No. Because like probably my I mean I would say you know you went through this once, I’m sure you can get over this again, you know you’re stronger and I believe that I could motivate the person much more than I did before.

I. Okay. Are there any factors that facilitate or help you to be compassionate towards others? Not just your mother in this scenario, but also the way you were brought up, any people or any circumstances that helped you to be a more compassionate person to other people?

P. So.. yeah one thing is my religion. Can I talk about religion in this?

I. Of course, and that is something I expect to discuss as well.

P. Okay (laughs). So.. In my religion which is Christianity, we are always taught that we should show compassion to others and it is something that you grow with. It is not something you should do, but it is something you should get from within you. And how you get compassion within you is following the religion, like say reading the bible, you know studying the religion, following it. And umm.. as you go on you will also feel like to show compassion you may not put an effort, it is something that comes out of you automatically with time. So yeah that is one factor and another factor could be my family background. I have seen from my small days, my parents showed compassion to strangers, say beggars by the subway. You know they used to maybe by them dinner and stuff like that.

I. Are there any barriers that make it difficult to be compassionate towards others?

P. Umm.. I don't think there's any barriers so far, but yeah. Ahh but then there's this thing, like say when you walk on the road. It's on casual day when you want to show compassion, you also feel insecure or you doubt if the, specially in Sri Lanka like you can't say, you can't trust anybody easily. So say you show compassion, we don't know how the person will take it right? So some people would like to be helped or may not like to be helped, or some people wouldn't like to be shown compassion maybe.

I. Yeah. Great! So you've told me about how you are compassionate towards other people. Now we're going to talk about how others show compassion to you. Do you need a break?

P. Can I?

I. Yes of course.

****Continues after the break***

I. I am now going to ask you to think about another time, this time when you were going through a difficult situation. It could be one or multiple situations. Can you tell me if anyone showed compassion towards you?

P. Yeah they did. There's a lot mostly my friends, they showed compassion. Can I specify the event?

I. You can, yeah.

P. So, that’s when umm.. I did not get the expected results I wanted for my exam. They were good results, but not to an extent I expected. So.. it broke me a lot and, my friends knew it. My friends too knew what my expectation was. And.. they yeah they were they showed me compassion like in the sense they called me on and off, they checked in how I was doing and they were with me and they organised outings, like they said let’s go out you know, now that the exams are over you know you can’t change anything. Both in actions and words they did a really big thing for me.

I. Why do you think that was?

P. Umm.. (laughs) because they love me. And.. I’m pretty sure like they may have understood who I was inside and like they really gave me what I wanted at that time. So there are some friends they know how to show compassion but it doesn’t address the centre of the how you say like, most of the time when people show compassion, it doesn’t directly go to the place where it needs to be gone. Say you want love like say you want yourself to believe in you, but like some friends are able to do it and some friends are not. There are certain specific friends who can do it, but not all friends can do it. (unclear) Am I going out of the question?

I. Absolutely not. I’m enjoying every bit of your answers. You said that they took you out and things like that. Are there any other things that you can recall which they did?

P. Umm they tried a lot to drift my attention from the problem. So yeah that’s what they did, like we went to exhibitions, we went on a lot of outings and they cracked, they cracked a lot of jokes. And.. yeah.

I. Okay.. What were your feelings and thoughts towards them?

P. So there was this point, at that time I could see some of the good characteristics in my friends that I couldn’t see before. Yeah. So say l have known this friend like for the last five years, but then the things I had not got from her for the last five years, I could see.. I could you know see those characteristics in her or say them at that time towards me. So I also felt like I was meant to have them.

I. That’s nice. And then afterwards…. How did their words and actions affect you? Did it help in anyway or did it make it worse or how did it affect you?

P. No they did. Their actions, words helped me a lot. My sadness and.. and.. tension reduced. I could be the happy girl they wanted me to be. So it did not take so many days for me to recover. Within a few days say like a week, I was totally recovered.

I. Sounds like it really did affect in a positive way.

P. Yeah positive way.

I. How about them - How do you think it made them feel?

P. Ummm.. I think. I think that they helping me must have made them feel good or and made them feel ummm…made them feel good to themselves in the sense that they may have felt, yes I did something for her that their attention, their words did positively affected me at the end. So.. they too may have had a self-satisfaction out of it.

I. If the same thing happened which again I don't hope for you, would you want them to do and say the same things?

P. I don't think that I would be so depressed say, like I did before. Because one thing that I have been through it once and I have learned many things out of it and knowing that there'll be people to comfort me, I don't think that I will say fall so deep and yeah I would expect them to be there.

I. Ok. Are there any factors that facilitate or help others to be compassionate towards you?

P. Yes.

I. What kind of factors?

P. There family background, how they are brought up and. How they are brought up in the sense from small days, if … if one can know the importance of compassion towards themselves and to others, like I said before it would not they would not have to put an effort to show compassion. It is something that you get from within you, so yeah.

I. Do you think that you as a person also has an impact on how they would approach the situation show compassion to you?

P. Sorry?

I. So we spoke about what things facilitate them to be compassionate people, but for them to show compassionate to you, are their qualities in you also as a person also that would facilitate their compassion to you?

P. Yeah yeah. Umm.. my qualities say if I am good to them, there's a bigger chance they show a lot of compassion and they would not think twice to show compassion, and if there are instances that they can remember that anytime I was not good to them or maybe harsh to

them. So there are some friends who would think twice before showing compassion. To some quota they would play or some limit they would place to show compassion.

I. So a person's personal qualities also would matter? Okay. Are there any barriers that make it difficult for others to be compassionate towards you?

P. No.. I don't see any barriers.

I. We are on to the third aspect now, which is about how we are compassionate towards ourselves. This time I am going to ask you to continue thinking about occasions when you were going through a difficult situation. Do you think that you were compassionate towards yourself?

P. I was in my experience no? So yes I believe like when you're in a difficult situation, when you want to cry, that is when you indirectly show compassion towards yourself. like you want to let it out right? So if you can go inside a room and then lock up and cry on your own, I believe that it is an act of showing self-compassion towards you. And if you are not going to open up to yourself and if you're going to supress any sadness inside you, so that is not showing self-compassion towards you and.. umm.. yeah.

I. Why do you think you were showing compassion to yourself?

P. It is with time that I have learned that it is important to love yourself, it is important to treat yourself, it is important to say nice things to yourself. So yeah it is something that I have learned with time. And with time I have learned, in order to show compassion towards others, you should have something inside yourself and that is compassion to yourself.

I. Okay. This is an extra question. But do you find it easier or difficult to show compassion to yourself when compared to showing compassion to other people?

P. No. I'm a person who always show compassion to myself, like in the form of dressing, even when eating, even when sleeping like I would say that I am a little selfish to myself. Like I want myself to be good, taken care of.

I. Ok. Wonderful. What were your thoughts and feelings towards yourself?

P. Umm.. so sometimes I got this, I used to think is it the right thing to do? Like is it needed like is it always okay to love yourself so much? So, that's one doubt I still even have. Yeah.

I. Any other feelings I don't know such as judgmental, kind or any feelings you get while being self-compassionate?

P. No. I love being compassionate towards myself. I feel happy when I know that I love myself. Because I know people and I see people who forget to love themselves and, but where as I always keep in mind it is important to love yourself, treat yourself, it is okay to look good, it is okay to try to look good so I don’t think it’s judgmental.

I. So it makes you happy when you’re loving yourself. Ummm.. How did you treat yourself in the sense what did you do to look after yourself when you were going through something difficult?

P. So when I went through some difficult situations, I try.. It is something that I practice even now like, say if I see imperfections in life? Say if there’s if there is something that doesn’t go right? I always try to see the even the littlest positive things in life, even the littlest thing that makes me happy I try to engage in that so that I would make myself feel happy. So say even say during exams, you can be caught up in studying assignments and all but then I always remind myself you know it is also it is needed to show yourself love, and how I do that is go to my momma go to my brother and watch TV with them. So, that’s how I show love to myself. And even like say how you dress? That is one big aspect in life that I follow.

I. I think dressing can actually make an impact in the way you feel. Were there any consequences for you from treating yourself the way you did?

P. I don’t remember any consequences like that.

I. Was there any impact? Say because your answers were very positive ones, so let’s think if you weren’t being as compassionate, would it have changed the way you felt or?

P. If I had not been compassionate to myself, there would have been my family or my friends noticing me and they may have come up to me saying no you shouldn’t do that to yourself, you should be like this be like that, so that is what has made me like to you know always remind myself to be good to yourself and there’ll be my mother, my father, my brother, my friends always reminding me no you are good enough, you are deserving enough to you know have this thing in life, that thing in life like that.

I. If the same thing happened again, would you do and think the same things about yourself or would you treat yourself differently?

P. I would continue to love myself and I would I would go in search of any new things I can do to prove that I love myself or to improve my love to myself.

I. Why?

P. Because I don’t know it’s something I believe that you should love yourself, you should love the life you have, you should love the image, you should love everything you currently have in life. So then you can look life in a positive way, look at life in a positive way.

I. Are there any factors that facilitate or help you to be compassionate towards yourself?

P. So like I said my religion. So that is what I have learned from my smaller days and my family and umm.. so ultimately the person that I have turned into will always show compassion to and show compassion to myself.

I. Would you say those are the childhood experiences or are those the things that you learned along the way?

P. It’s both. Childhood experiences plus what I have learned along the way. And talking about my childhood experiences, I had a very happy childhood. Like I remember mostly I spent my time at the day care after school, so I had so many friends, teachers were friendly and parents were I mean parents were good. And everything was friendly, I don’t specifically remember any incident that would have changed me, so.. I believe it’s it’s later in life like later in my teenage life, many of the negative things happened. So even in those times I believe like the positive characteristics in me have helped me deal with them and through that maybe show love to myself.

I. Final question. Are there any barriers that make it difficult to be compassionate towards yourself?

P. Umm.. I wouldn’t be too selfish like even if I’m selfish to myself I wouldn’t be too selfish. So I wouldn’t be too selfish that’s a fact I believe I can..

I. Ok but are there any other things that have stopped you from being nice or kind to yourself?

P. Yeah so when I’m angry (laughs) there’s this time that I feel that I need not have love or maybe not show love to yourself. That’s what I feel when I’m angry at myself or someone for something, then I tend forget to like treat yourself, you know like I skip meals. That doesn’t happen often maybe like once in a while, so yeah I believe that, that is some barrier.

I. That is the end of the interview, do you have any questions?

P. No.

Interview 5.

I. So for the first question…. Can you tell me what the term compassion means to you? So when you hear the word compassion, what does it make you think of?

P. Feelings towards people, yeah sympathy, love. I think it is all those things when they are suffering and like being there for them you know.

I. Can you talk about your understanding of self-compassion?

P. As in?

I. In the first one I asked generally about compassion. Now we put a word self in front of the term compassion, referring to the compassion that you give to yourself. What does the term self-compassion make you think of?

P. Ah yeah. Then I guess it's giving yourself self-love, making sure you are okay when you are hit with problems and failure.

I. Absolutely. The next set of questions are going to focus on three aspects of compassion. The first one is how we show compassion to other people, the second is how others show compassion to us, and the third is how we are compassionate towards ourselves. So for the first one, I'd like you to think about one or two occasions when a loved one was going through a tough time or difficult situation. This could be a family member or close friend and could be when they were sick, failed an exam, lost a loved one or anything that upset them. You don't tell me what the situation was or who the person was. Can you tell me if you showed compassion towards them?

P. Yeah

I. Could you tell me why?

P. Because I love them and care about them.

I. Can you elaborate a little bit more? Do you think they needed you?

P. Yes.

I. Can you tell me the things that you did or said to them?

P. Can I tell the thing? When my dad got sick, umm.. I did everything for him to be comfortable and to be to umm.. make him feel that we are with him. Because he got a heart-attack and almost went to..

I. Ok. So as you said you tried everything in your capacity to make him feel good, right?

P. Yes

I. What were your feelings and thoughts towards him? How did you feel about him?

P. I felt really bad. Because I never expected it. And when he got it yeah.. and I never talk about that.

I. You really don't have to talk about it if you don't want to and if you want to change the situation to something less stressful, you can also think of something less stressful ok. And then talk about that if you if you prefer to talk about something else instead of this one.

P. Ok I lost my grandfather, that was..

I. Ok. So you can you can, the point is you can talk about anything. It could even be something way less stressful like a friend who was having a relationship issue or something like that. You can think of anything, but the point is to understand how you felt for the person and how how and if you actually did something to change their situation. So you can think of all these. You can either think of one particular situation or you can think of all as a whole and answer my questions. So, you said you were feeling really bad. And then after, after you showed compassion how did your words and actions affect them?

P. I don't know. I can't remember that.

I. Yeah. Can you think of maybe some other situation?

P. Yeah my, one of my best friends went through a bad breakup. And yeah, I talked to her a lot and even recommended a relationship counsellor. So they felt she felt very good about me talking to her and she could even move on. So.

I. Ok. Wonderful. How about you? After being there for her and helping her, her or him, was there any impact on you?

P. Umm.. yeah even I learned things about relationships. I am in a relationship and I got scared too. But yeah.

I. Okay. Umm.. How did it make you feel after being there for her knowing that you were there for her.

P. I was I felt good.

I. About yourself?

P. Yeah.

I. OK. If the same thing happened again, to this friend or another friend, would you do and say the same things or would you react differently?

P. Maybe it will depend on the on their situation, what kind of situation. And yeah.. Kind of situation they are going through, if it is hard I'll be there, like I want to. If it's like an easy one I won't be recommending a relationship counsellor, I'll talk to them.

I. OK. Are there any factors that facilitate or help you to be compassionate towards other people? I'll make the question a little bit easier. So.. you know when we grow up there's our religion, our upbringing, things our parents teach us, and sometimes some people that we learn from, like the role models in our life. So for you in your life, are there any factors, people or experiences that have made you a compassionate person towards other people?

P. Yeah. My mother and my parents, and yeah religion. From religion we learn a lot.

I. Can you elaborated a little bit more on how how these things have taught you?

P. Umm.. so my mother and me are very friendly. We talk, I tell everything to her. And she's also not that strict. My parents are very (laughs) my parents are comfortable. So I learn things from them and also my boyfriend, yes. He he was the one, he's a bit religious and made me religious and that made me more compassionate about others and myself as well, yeah.

I. So do you think having people that influence you does make an impact on how somebody shows compassion to another person?

P. Yeah also maybe can be.. yes.

I. Okay. Umm.. same way are there any barriers that make it difficult to be compassionate towards others?

P. Umm..

I. Do you think there are some factors or sometimes some people or some experiences that sometimes stop you from being very compassionate to some some people.

P. No. Okay, I said I'm a bit religious. So.. being kind is a the main thing. So I don't hate people, I really don't hate people. Even friends they can be stupid (laughs) ok.. but I don't hate people. So if they need any help any kind of help I would help them with anything. Even if it's a stranger, I would. Sometimes.. sometimes.. umm helping stops when there's a lot of people around and I'm a bit shy too..

I. Ok. Yes, yes that's what I was looking for. Because we all have sometimes I can be a very compassionate person, but if I'm anxious to show when there are people around, I get uncomfortable so I step back. So are there any other things or situations that sometimes stop you from showing compassion that's in you?

P. Yeah. Those kind of. Because in crowds I get I get I don't know if it's a phobia (laughs). Yeah then I stop that and umm.. yeah.

I. Yeah, same with me. I am comfortable doing this interview. But if there were 100 more people the situation would be different. So it's a common thing for all of us (laughs). Ok. So you've told me about how you are compassionate towards other people. Now we're going to talk about how others show compassion to you. I am now going to ask you to think about another time – when you were going through a difficult situation, not somebody else. Can you tell me if anyone showed compassion towards you?

P. Mmm.. yea

I. Why do you think that was?

P. So it's my mother. So she loves me. Maybe that's why. I am close to her, and I don't talk personal things with everyone, not even with my friends sometimes. So it's my mother always and boyfriend and some friends. And yeah they showed compassion to me.

I. Can you tell me the things that they did or said to you that made you feel like oh they were actually being compassionate this time?

P. Umm.. so this time umm… okay weeks back my boyfriend.. my boyfriend studies in UK. (unclear). And he came he's coming every year one or for two months around and when he's leaving it's really hard for me. His parents and my parents they were comforting me. His mother was like you are like my own daughter, you come here anytime you want. (laughs) Yeah she's sweet and my mother also, she was like joking around to cheer me up. Yeah.

I. Ok. Umm.. so I guess one way they dealt with it is by saying you have us, you can come to us anytime and also by joking about it so you feel good.

P. Yeah he will be back in another 10 months, yeah.

I. Ok. What were your feelings and thoughts towards them?

P. I felt really good and I really love them.

I. Any thoughts about them? Did you think..or they are the best or something like that?

P. Yeah they are the best. Yeah they are so sweet and even if I break up with my boyfriend I would really go to his parents (laughs) (other voices)..

I. And then afterwards…. How did their words and actions affect you? I mean you answered it but anything you want to add to it? You said you felt really good and?

P. Yeah I felt really loved.

I. How about them - How did it make them feel? Do you think when they were comforting you did they feel good too or did they feel sad or how do you think?

P. They felt good and sad, she also cried with me (laughs).

I. Aww okay. Any other impact or any other outcome from it?

P. Ummm.. yeah that's about it.

I. Umm.. If the same thing happened again, maybe the next time he comes and leaves or you can think of a different situation, would you want them to do and say the same things? I will just help with that question, cause sometimes let's say I go through a breakup or something and somebody comes and talks to me, my friends keep telling me you have to move on and this and that. Sometimes it helps because you want someone to talk to me, you want someone to take me out and make me feel good, but another time it happens, I may feel like I need some personal space, I want them to step back, so think about these situations that you were going through a hard time. It could be when your boyfriend left or it could be some other situations as well and when people were there for you maybe you felt good. But if it happens again, would you still want them to be there and do and say the same things or do you expect a different reaction from them?

P. Umm.. I would expect the same things and even I don't mind different things because I love them and they love me. It's about parents and they are really sweet.

I. And I think in this situation it makes sense to expect the same things right? (laughs) Okay. Are there any factors that facilitate or help others to be compassionate to you? It's like the same question we discussed before. You spoke about your religion.. Do you think there are any factors that have made people the compassionate people they are?

P. Looking religiously?

I. Could be anything, it could be religion, it could be the way they were brought up, could be their school or the people they associate. Anything you can think of that have helped them to show compassionate to you?

P. Can you repeat the question?

I. Yes. So.. umm your mother, your boyfriend’s mother those people you think are very compassionate people. They are very kind, when someone goes through something they talk to you, they comfort you, they cry with you. So all these things show that they are very compassionate people. So what do you think have made them so kind and compassionate people?

P. Yeah.. their religion.. and maybe ummm.. how they were raised as well. Yeah they had loving parents, they must have got attention even my parents.

I. Exactly. Like for you you said that you are very close to your mom and father so those things make you the kind person you are so the same way for them. Are there any barriers that make it difficult for others to be compassionate towards you? Do you think there are any factors in their life sometimes make it difficult for them to be compassionate? Or even make it difficult to be compassionate to you as a person?

P. Maybe being a parent. Ummm… I don’t know.

I. So for example for me. Sometimes my mom can be right there with me helping me. But if I say I want to be alone, even if she wants to be compassionate and helpful, sometimes she can’t because I want to stay away and I’m refusing the help. It’s just an example. But are there any thing like that as a person that you have that make it difficult for them to reach out and be compassionate to you?

P. No they are like very comfortable with me.

I. And you like the comfort you get from them?

P. Yeah.

I. OK. We are on to the third aspect now, which is about how we are compassionate towards ourselves. Now you showing compassion to you. This time I am going to ask you to continue thinking about occasions when you were going through a difficult situation. Do you think that you were compassionate towards yourself?

P. Yeah.

I. Why do you think that was?

P. Mmm.. because seeing how other people going through bad situations, even I don’t want to get into them and be (unclear).. and umm.. I can be resilient.. yeah and I think again I don’t know how to explain (laughs).

I. It’s okay. Umm.. Do you want me to go to the next question?

P. Umm.. wait I’ll try to answer.

I. Yeah take your time. I’m not in a rush.

P. (laughs) What’s the question again?

I. So you think you are a compassionate person to yourself as well? You said you are compassionate to others when they were going through a tough situation. Now I asked are you a compassionate person to yourself when you are going through a tough situation? If so, why? Why do you show compassion to yourself?

P. Yeah.. to to make myself feel good yeah. To have a good life?

I. Yeah to help with the situation?

P. Yeah.

I. What were your thoughts and feelings towards yourself? Think of a difficult situation you faced and when you were showing compassion to yourself, what things did you feel and how did you feel about yourself when you were going through this tough time?

P. I felt good I mean this time it was when I lost my grandfather. I was really sad, but then I started to feel that okay everyone dies and being sad is not the best thing for them as well as for me. Yeah..

I. How did you treat yourself? Now you said you thought this is a common thing and you are not the only one. Are there any other things you did to look after yourself? It could be eating healthy food or anything that you did to show that you were actually taking care of yourself?

P. Ah yeah I eat healthy. My whole family is a healthy family and we eat healthy. Yeah we eat healthy and..

I. Did you continue the normal day today things you did or after the passing of your grandfather did you stop doing the normal things or did you?

P. No I did my normal things, yeah.

I. Okay. Were there any consequences for you or any impact for you from treating yourself the way you did? It could be positive, it could be negative. So when you were being kind to yourself, how did it result? How did it address the issue?

P. It was a positive result.

I. You felt good after being kind to yourself?

P. yeah.

I. If the same thing happened again, not necessarily the same event but any.. any any situation where you were going through a sad or upsetting thing, would you do and think the same things about yourself or would you treat yourself differently?

P. Again it will depend on the situation and mostly I try to understand the living thing the system and make myself better feel better and also others feel better. So even my family everything we are like there's nothing sad to talk about. We are a happy family so I give them advice and I get advice very well. So from that also I try to understand the life very well. So..

I. Again similar to the previous question. Are there any factors that facilitate or help you to be compassionate towards yourself?

P. Yeah same answer. My parents, my religion and everyone that I care about, everyone who loves me.

I. Can you explain how your religion has helped you to become a compassionate person?

P. Umm.. it's more the buddhis Buddhism is mostly about not doing sinful things, being kind to everyone, meditation.. it helps a lot.

I. Do you practice meditation?

P. Yeah.

I. How often?

P. Umm.. once a month on poya days, I observe sil.

I. Oh that's wonderful. Ok again Are there any barriers that make it difficult to be compassionate towards yourself?

P. Ok I also meditate when I'm having umm.. umm when I want to feel good, at that times also.

I. Ok final question, anything that makes it difficult to show compassion to yourself? You know sometimes we go through really tough times, sometimes when everything seems to be going wrong, it's hard to be kind you know what I mean? Are there any situations like that where you have felt like sometimes it's hard to show compassion to yourself?

P. Ummm.. I can't really think of anything. As I said I have a really really helping comfortable family and all. Sometimes I do change my diet when I'm angry.

I. Okay. That's a very good example. Can you explain how you change your diet?

P. I starve. I starve myself. For a day sometime.

I. Okay that's the end of the interview, do you have any questions?

P. Umm.. I don't know. Can I ask you what you feel about compassion?

I. Okay I'll save the interview and then let's talk.

P. Okay.

Interview 6.

I. So for the first question…. Can you tell me what the term compassion means to you?

P. Umm.. Compassion is a gifted thing. I think ummm.. it's something gifted and it's it's a needed human quality.

I. Ok.. Umm.. can you elaborate a little bit more as to what qualities you have to have to be compassionate?

P. Yeah. I've seen this quality in people have a thing called emotional intelligence, ok. People with high emotional intelligence levels, they have that quality called compassion, ok. And being compassion is something gifted as I said before and.. in the modern world I think compassion is.. kind of an artificial thing. That's it.

I. Ok now we spoke about what compassion is in general. Can you talk about your understanding of self-compassion?

P. Ummm.. self-compassion. It.. it is kind of a compassion that is towards you and it is very essential because if someone has a greater level of compassion towards yourself, that person is is kind of a very nice person, and yeah.

I. Brilliant. So.. The next set of questions are going to focus on three aspects of compassion. The first one is how we show compassion to other people, the second is how others show compassion to us, and the third is how we are compassionate towards ourselves. So for the first one I'd like you to think about one or two occasions when a loved one was going through a tough time or difficult situation. this could be a family member or close friend maybe when they were sick, failed an exam, lost a loved one or anything that upset them. Can you tell me if you showed compassion towards them?

P. Yeah I definitely showed compassion towards them and and I give too much. Actually when I show my compassion to someone, I become too much, yeah.

I. Could you tell me why?

P. Yeah. Later I realised that is my weakness, one of my weaknesses. So.. but I can't control myself when I become compassionate, I get truly attached to that person's emotions, ok? And I see things according to that person's view and get that problem to make my my problem, ok. And.. that's it.

I. Can you think about the things that you did or said to them which you think were being compassionate?

P. Yeah.. I actually tell so many stories on behalf of them. Actually I'm going to I don't know.. I use lot of my personal experiences to umm.. give them kind of a relief, ok. And.. umm.. sometimes they are really really sad. When I see that I become too emotional and I want that person to be happy, so sometimes I lie because I want that person to be happy and get rid of that discomfort. So I do anything I can.

I. Ok.. Umm.. When someone you like was going through a difficult situation what were your feelings and thoughts towards them?

P. Like.. umm.. I feel really compassionate towards them and I get sad and umm.. I I.. tend to be more kind towards them and umm.. I think umm.. what if this person has no one to help, there's no one to help this person so what is going to happen to that person, like that.

I. Ok. Brilliant. And then afterwards…. How did your words and actions affect them?

P. Yeah definitely I can see results after showing my compassion. I.. most of the time I talk to those people. I talk with them and I become so friendly with them and I can see results quick. They become kind of happy and umm.. sometimes most of the time they told me that now I'm a little bit relieved I feel kind of comfortable umm.. like that so I get happy because they are happy.

I. How about you? Was there an impact on you after being compassionate? Good or bad could be either.

P. I have so many experiences umm I'm not going to talk about them now. Umm.. most of the time at the end of the day I'm the worse person in this world, to them.

I. That's how you feel?

P. To them. To them because with their reactions I can understand that according to them I'm the bad person. Most of the times, ok. But.. umm. Yeah.

I. Okay. Because you said that the impact was that it ended up being that you tried to be the helpful person but it ended up them thinking you as the bad person. If the same thing happened again, would you do and say the same things?

P. Umm.. I think I think it basically depends on umm.. the person's personality and their thinking patterns. And.. I wouldn't change the way I show my compassion towards someone because I know it is the most affectionate way to show someone. Yeah it would happen.

I. You would still go and be the compassionate person you are?

P. Yeah.

I. Ok. Umm.. Are there any factors that facilitate or help you to be compassionate towards others? Umm.. this could be circumstances, people, good influences bad influences I don't know. Anything that have shaped and helped you to show compassion to others?

P. As I was raised in a Buddhist family, we we've been teaching how to be compassionate to people. And.. ummm I actually I I don't expect someone to react in a good way after I show them compassion. I don't expect anything. Umm.. I.. for me being compassionate to someone it gives me kind of a relief. And I get satisfied, I become really happy oh I did I really helped that person, I showed compassion, so that person is happy, I'm happy. That's why actually I use it as a stress relief. I'm stressed and yeah.. that's it.

I. Are there any barriers that make it difficult to be compassionate towards others?

P. Yeah.

I. Can you tell me what those are?

P. Umm.. most of the time social barriers. Umm.. and yeah. Social barriers and umm.. people's personality, their levels of understanding something. People who need help,

sometimes they can't understand that I'm trying to help. Sometimes they misunderstand. But I truly want to help them, I truly want. Social barriers and their own personalities.

I. So what are the social barriers?

P. You know in Sri Lanka, when I try to help a girl, people see it as a different thing. My parents too. And there are so many friends who comes to me when they need help, mostly girls. My parents sometimes misunderstand that I have many girlfriends. But that's not true. I want to help them. And.. if we consider about other people, I mean the society, they see it as a real different thing. Because they are in a frame called tradition, yeah.

I. It is very interesting. Would you say tradition and would you call it people judging you are barriers towards becoming a compassionate person?

P. Yeah.

I. That is very interesting. Great! So you've told me about how you are compassionate towards other people. Now we're going to talk about how others show compassion to you.

P. Okay.

I. I am now going to ask you to think about another time – when you were going through a difficult situation or two. Again this could be when you were sick, failed an exam, lost a loved one or anything that upset you. Can you tell me if anyone showed compassion towards you?

P. Yes

I. Okay. Why do you think that was?

P. Umm.. ummm.. some people actually wanted me to get rid of that stressful situation. But some.. they have kind of expectations umm.. yeah.

I. Okay. Expectations as in personal expectations for them or?

P. Yeah personal expectations for them, for them.

I. Okay. Can you tell me the things that they did or said to you that made you think oh they're being compassionate to me?

P. Those people?

I. Yeah.

P. Ummm.. Their compassion… actually that's a fake thing. I feel that. They say oh just just ignore that, you're a good person, don't try to be too emotional, when we're in a situation you're the one who help us, they say so. But I think it's because I help them, it's not because they want me to be happy. They sometimes they fake. I feel that umm… and they just say just ignore that but they.. when I meet them they tell me ohh.. but then they don't they don't pay much attention to my problem, ok. I need.. you know when someone is in a problem, that person needs kind of attention for a time, but they just say they just say ah ignore that, you're a good person and say blah blah blah.

I. So is everyone like that or are there other people who..

P. No. As a percentage 20% people are like that and 80% are good.

I. So they really want to help you?

P. Yeah

I. Okay. What were your feelings and thoughts towards them?

P. I understand that you mean those people right?.

I. So. Yeah you can tell me about the person who actually wanted to help you as well as the people who were faking. So how did you feel about them when they were showing you compassion?

P. Fake people?

I. Both. You can talk separately about both.

P. Ah okay. So fake ones, I feel I don't actually most of the time I am I become a counsellor for myself. I listen to them, I listen to their ideas and umm.. take their opinions and think about them and umm.. I umm.. I become a counsellor to myself and I get out of that situation somehow. And I feel yeah these people are trying to help me, they are showing compassion towards me but not like the way I do.. umm like that.

I. So do you feel happy or when you realise that they are actually being fake. How do you feel? Would you still want them to be compassionate or would you want them to just stay away from your problems?

P. Umm.. actually I don't care, because I don't expect someone to be compassionate towards me. Most of the times I have received so many bad experiences from being compassionate to

someone. And and I have my personal experiences so I can make my umm.. I can make my umm.. I can make my mind myself. So I don’t want them to be I don’t want them to be compassionate towards me.

I. And then afterwards…. How did their words and actions affect you? Did it make any difference to the problem or the way you felt?

P. Yeah kind of. As I said before, I think about their opinions when I was in bad moods, and just try to make my mind. Not too much, I’m not thinking about them too much.

I. Okay. How about them – How do you think them showing compassion to you affected them?

P. Umm.. fake people may think ah okay okay he become compassionate towards us when we are in bad mood, okay now we did our duty, they might think like that. But good people, they might feel as the same as me. I think they get a kind of a happiness by showing their compassion to me.

I. Okay. If the same thing happened again, would you want the same people to do and say the same things in terms of showing compassionate towards you?

P. Ummm.. Yeah, sometimes.

I. Can you explain why?

P. Yeah. As I told you before I can make my mind myself. But sometimes with their influences, ummm.. they have kind of a influence of influence on my thoughts to become more helpful, okay? And.. yeah.

I. Are there any factors that facilitate or help others to be compassionate towards you? Do you think that there are any factors that have made them wanting to be compassionate to you?

P. Compassionate to me?

I. Yeah.

P. (laughs).. ummm because they are they are friendly people to me. And they are they might be sensitive and umm.. they may like me.

I. Could it also be because when they were going through tough situations you were showing compassion, so they feel compelled to help you when you were going through something?

P. Definitely, that would be a reason.

I. Are there any barriers that make it difficult for others to be compassionate towards you?

P. Yeah as I told you before, social barriers and but the only barrier is social barriers. Because when someone is trying to help me, I'm not going to see them as a in a very narrow mind. So that problem won't arise. Yeah.

I. Okay. So you think just like social barriers were affecting you they also affect these people when it comes to showing compassion to you?

P. Yeah

I. We are on to the third aspect now, which is about how we are compassionate towards ourselves. So there will be no social barriers I guess, we can we can talk about it later. This time I am going to ask you to continue thinking about occasions when you were going through a difficult situation.

P. okay.

I. Do you think that you were compassionate towards yourself?

P. Yeah, definitely

I. Why?

P. Because I love myself and umm.. I love my weaknesses, I have my strengths. Umm.. my personality, my soul I love it. I want it to be helped, I want it to be happy, so yeah the only reason is I love myself.

I. What were your thoughts and feelings towards yourself when you were going through difficult times?

P. Most of the time, I have felt that umm.. umm.. I talk to myself. Umm.. it says look at you, you have no one, sometimes you have no one like you were for others when when people are in a bad mood, I used to go and help them, I used to go and ask are you okay and fully check with them and help them. But to me, not like that, people don't come towards me. So.. I feel a little bit sad about myself sometimes, but I become so strength via that. And.. I strengthen my thoughts and try to get rid of that stressful situation somehow.

I. How did you treat yourself when you were going through these tough situations and when you were having these sad feelings towards yourself?

P. ummm.. I treat myself nicely. Umm.. saying myself that you are a good person, you have a good mind, you help people so in return you will receive something, ummm..and don't be sad, this is just a temporary situation, just let it go, try to try to cope with that as you can and umm.. just be happy.

I. OK. So in terms of actions other than talking, I don't know in terms of going out for a movie or something or diet or anything. Was there any change or is there anything that you did to treat yourself in a good way or bad way, could be either.

P. Yeah. Mostly the guitar, the guitar.

I. Oh ok you play guitar? Wonderful.

P. Yeah, guitar is my best friend. I actually can't even think of living without that instrument. Whenever I feel sad, stressed or bad feelings, I'm just going towards my guitar and take it and play it in a dark place.

I. In a dark place?

P. Yeah.

I. Is there a particular reason as to why you go to a dark place?

P. Umm.. I love dark. I love darkness and the feeling I get when I play in the darkness, it's it's unexplainable.

I. Okay. So is it a positive feeling, is it a calming feeling?

P. It is a massive feeling, it can heal everything. As I think, it can heal everything and it is kind of a best feeling that I have felt.

I. Okay.. Were there any consequences for you from treating yourself the way you did? And what were they? So when you were showing compassion, or maybe at times when you weren't. So when you were going through tough times, from the way you treated yourself, were there any consequences for you?

P. Yeah. Umm.. so far.. but.. umm.. but sometimes I have I have neglected some duties that I was supposed to do in.. in order to get rid of that stressful situation. Umm.. not most of the times, rarely I have done that. Umm.. so as a result of that I… there have been so many disadvantages.

I. Can you elaborate a little bit more, how did you neglect yourself and what were the disadvantages?

P. Umm.. like people have understood myself as a as a.. how do you say as a different person. I mean as a bad person, sometimes they have understood like that. And.. as a result of that thought they have been they have showed me kind of a rudeness sometimes and ummm.. so I feel guilty most of the times when I have to face such a situation.

I. Okay. If the same thing happened again, would you do and think and say the same things about yourself? Or would it be different?

P. It depends on the situation, but yeah maybe.

I. Ok. Would there be any other different ways that you would approach yourself or would it be similar to the way you have been dealing with yourself in the past

P. You mean?

I. When you go through something difficult?

P. No actually the same way.

I. Okay.. Are there any factors that facilitate or help you to be a compassionate person towards yourself? And if so, what are they?

P. It's basically I love myself and umm.. and yeah I want I want to help other people. So, I'm supposed to be a good person to myself. That's the reason.

I. you previously in my first question, you also said about your Buddhist environment. So does that also have an impact on the way you treat yourself?

P. Yeah. Yeah definitely. Because I respect Lord Buddha's philosophy, and he's a very compassionate person. And even when we consider about Jesus, Jesus is also a person with high compassionate level. And being compassion is something gifted, it is something very nice. It is a very nice quality, it is it is a good human qualities.

I. So when you think about the way you show compassion to other people, when they are going through difficult situations. Is it similar the way you treat yourself or is there a difference in the level of compassion you show to yourself and the level of compassion you give someone else.

P. That's what I said. I become too much. Too much means, I equally umm… show compassion to others and me as well. Okay? No difference.

I. So there’s the same level.

P. Same level.

I. Are there any barriers that make it difficult to be compassionate towards yourself?

P. No.

I. So you allow yourself to be compassionate?

P. Yeah.

I. That’s it. I’m going to save the recording.

Interview 7.

I. So for the first question…. Can you tell me what the term compassion means to you?

P. I actually don’t know.

I. That’s fine. Can you think about the, when you hear the term-compassion, what kind of feelings do you get, can you think of any maybe similar words that comes to your mind when you hear the term compassion?

P. Maybe it’s about yourself. I always get that that word usually goes with yourself.

I. OK. It’s interesting that you said yourself, because my next question is can you talk about your understanding of self-compassion?

P. So, what do you.. Can you please define the word compassion?

I. Ok. So.. in the study that’s what I am trying to see if people understand. But to give you an idea, compassion is about when something goes wrong, understanding that this exists, you’re not the only person who’s going through something like this whatever the situation is, number two acknowledging something like this is happening, so it’s not like you’re completely denying that you’re going through a difficult situation, you acknowledge you are mindful about the situation, but you accept it in a non-judgmental and kind way. Third one is kindness, so whatever that may come on your way at your way, you are being kind about it rather than being critical and judgmental, you are being kind to yourself, okay?. Did you get an idea?

P. Yeah.

I. Now if I ask you again, what does the term compassion means to you?

P. It means, it doesn't like.. I don't do that to me. If I come across any situation which breaks me down or like any problem to me, I just blame myself. I don't like go with the flow or think that it's normal and all that stuff, I just blame myself for it.

I. Which is a very common human reaction to problems. Ok I'm going to repeat the next question. Can you tell me what your understanding of self-compassion is?

P. I think it's all about calming ourselves when we go through a big problem and then thinking it's okay, it's fine like every human being has to go through these stuff.

I. Brilliant. The next set of questions are going to focus on three aspects of compassion. The first one is how we show compassion to other people, the second is how others show compassion to us, and the third is how we are compassionate towards ourselves. So for the first one…I'd like you to think about one or two occasions when a loved one was going through a tough time or difficult situation. This could be a family member or close friend and could be when they were sick, failed an exam, lost a loved one or anything that upset them. Can you tell me if you showed compassion towards them?

P. Yes

I. Could you tell me why?

P. Because I felt they were left lonely and they had no one. Can I tell you the incident?

I. You can if you want to but if you're not comfortable don't have to.

P. Yeah so I had my friend in my class who was in a relationship for three long years and that guy like left her for no reason and at that situation everybody in my class blamed her saying like you're the one who has (unclear) or you're lying or you are doing a drama and everybody left her out. So I was with her and I felt she needs love, she needs support and someone who will understand her. So I was with her at that time like throughout 6 months and my own best friend left me for that.

I. Ohh okay. It's interesting that you gave me this answer. Can you tell me more about the things that you did and said to them in order to show compassion to them?

P. I was like you need to let go because maybe that guy doesn't deserve you like you did so much for him and he left you for no reason. So.. she was like very attracted for his looks so I

was like don't go for looks and all those stuff and somehow she calmed herself, she was okay somehow.

I. Okay.. What were your feelings and thoughts towards them when they were going through this situation?

P. I felt very like I was a bit depressed like thinking about her. She was always crying and I was like I don't have to love anyone here like I have to go through the same shit.

I. Olay.. And then afterwards…. How did your words and actions affect them?

P. Actually like she really like took all my words into a good thing and like she start acting on it and now she's really happy with all what I did to her and actually she knows what I told was right for her.

I. Ok. So.. Do you think your actions and words helped her?

P. Yes

I. How about you? Was there an impact on you from that incident or from being compassionate?

P. Yes. Many people blame me for like going and supporting her and talking to her. Many people ignored me, my best friend like totally ignored me she's not even talking to me and that affected me a lot and still it's affecting me.

I. Ok. This is really interesting because it had an impact on you but also it helped the person you were helping. So if the same thing happened again, would you do and say the same things?

P. Yes

I. So regardless of knowing that you could lose I don't know friendships and things like that, would you still go and do the same things?

P. I would say that if my friends are really my friends, they won't leave me for such silly stuff. So I would surely go and help her.

I. Okay. Are there any factors in your life such as people, like your friends, circumstances or anything that facilitate or help you to be compassionate towards others?

P. No.

I. You think there are no

P. Nobody

I. Ok. If I elaborate a little bit more, are there any factors like your background, your culture, your upbringing, the way your parents brought you up, your religion. Have any of those factors or anything related to that helped you to show compassion?

P. Yes.

I. Can you explain what?

P. Growing up, like my family is a very huge family. So my parents are very like down to earth persons. They don't care what others tell. They just go and help people. So they get ignored, they get bullied for doing that when it comes to helping. But still by looking at them I feel doing that is better.

I. Ok. Are there any barriers that make it difficult to be compassionate towards others?

P. Yes.

I. What are those?

P. My friends and my siblings and my ex boyfriend.

I. Okay. In what ways were they stopping you from showing compassion?

P. They were like you're not the godmother to go and explain people and make them understand. You can just be on your own, why do you have to worry about them go and talk to them like that.

I. Great! So you've told me about how you are compassionate towards other people. Now we're going to talk about how others show compassion to you. I am now going to ask you to think about another time – when you were going through a difficult situation or two. Again this could be when you were sick, failed an exam, lost a loved one or anything that upset you. Can you tell me if anyone showed compassion towards you?

P. Umm.. yes

I. Why do you think they may have shown you compassion?

P. I’ll tell you the story again.

I. Okay.

P. So.. I went through a breakup. It was a very bad breakup. I caught my boyfriend cheating on me for more than five times.

I. Ohh I’m sorry.

P. And then my parents are difficult parents who worry about what other people will think, so they got me engaged to a guy who I don’t know. So that guy was like I told him, I am already affected with that person and I am not ready to accept your thing. And he was like okay, I’ve gone through the same thing, so I’ll support you, I’ll be with you, I’ll be your best friend, I’ll take everything whatever you feel and I’ll share the feelings. So he was with me like he told me this is life, whatever happen to us and he like came a long way with me.

I. Ohh, that’s wonderful. But why do you think he was being compassionate to you?

P. Because he faced the same situation.

I. Yeah, so he knew what you were going through.

P. Yeah

I. Umm.. can you tell me, you told me a little bit but.. can you tell me more about the things that they did or said to you?

P. So.. he was not arrogant to me like my ex boy my ex boyfriend was very strict like kept giving me rules, he was ruling my life, so first of all he listened to all what my ex boyfriend did to me and he did the positive things to me. He let me have whatever I like and he gave me all the freedom that I actually wanted and totally made me forget that incident in a short period of time.

I. What were your feelings and thoughts towards him when he was showing you compassion?

P. I was like okay there’s someone for me who would show me love and understand me.

I. So you felt all positive feelings for them.

P. Yeah positive.

I. And then afterwards…. How did their words and actions affect you?

P. Like, it made me realise that life is not shit. Like it really made me realise it’s okay it’s not everything doesn’t rely on beauty or money. There’s something other than that as well.

I. Yeah.. How about them - How do you think their actions made them feel? So, when they showed compassion, it helped you? But how do you think it affected them in return?

P. Like I don’t think. Like they would have liked (unclear) me more. They know I’ve gone through this so they won’t hurt me.

I. If the same thing happened again, would you want them to do and say the same things?

P. Yes, maybe. Maybe because.. umm.. I’m the person who keeps my private life private. Just because of the research, I’m trying to help you out.

I. Also if you’re not comfortable sharing anything you don’t have to.

P. No no it’s fine, it’s nothing like that. I love helping people. So.. like I don’t show that I am down. I’ve always been down in a way, but I don’t want others to feel down because of me. I never want to add to anyone’s problems. Nobody should feel bad about me.

I. So.. because you don’t want to put them in a difficult situation, you maybe don’t expect them to show compassion to you?

P. Yeah.

I. Okay. Again.. Are there any factors that facilitate or help others to be compassionate towards you? Can you think of any things, situations maybe people that may have made them to be more compassion?

P. The culture, their family.

I. How does culture and family come into play?

P. Because there are some people like who don’t care of what others are going through, they don’t give a shit. But there are some people like who have been brought up that way. Their parents like how they do that way.

I. Are there any barriers that make it difficult for others to be compassionate towards you?

P. No, I don’t think so.

I. We are on to the third aspect now, which is about how we are compassionate towards ourselves. This time I am going to ask you to continue thinking about occasions when you were going through a difficult situation. Do you think that you were compassionate towards yourself?

P. No I wasn't

I. Why do you think that was?

P. Because I thought like when it comes to my ex boyfriend's story, I thought I wasn't good enough, that's the reason he chose five other girls instead of me like I was like always torturing myself like I don't look good, I don't like I'm not good at anything and that's the reason why people keep hurting me and going for another girl.

I. Now you showed compassion to other people when they were going through a difficult situation, other people showed you compassion and you accepted it. So why do you think there's a difference with you showing compassion to other people but not to yourself?

P. Because, I don't know. I always have this thing of not, I don't have positive feelings about me, I always have negative feelings about me like you're not good at this, you're not good at that I don't appreciate myself for anything. I barely appreciate myself. Because even if I do something great, my parents don't appreciate me. So now, I don't appreciate anything. I have difficult parents who worry about what others think, so they got me engaged to a guy who I don't know.. no point talking about that even.

I. So those experiences and feelings have kind of registered in your mind?

P. Yeah

I. Okay. You said a little bit about this like I'm not good enough and things like that. But are there any other thoughts and feelings that you had towards yourself?

P. Yeah. Like my body.. like I was a very you know if I think of something, I end up doing the thing somehow. I was very fat before, and I just came to size zero because I wanted to lose weight. But then I grew a bit fatter, my boyfriend like broke up with me for that reason. So that like stressed me out, no matter how much I want to lose weight I can't lose weight because that's still in my head.

I. How did you treat yourself?

P. Like.. I don’t know I shouldn’t have done this to me but I was really bad for myself like I.. I feel so guilty for doing that to me. I shouldn’t have cared about all these stuff. I should have like thought that thank god I came out of this shit and such toxic relationship. But it was a really bad thing I did to myself telling that it’s all my mistake, it’s all my this thing that it shouldn’t have been that way.

I. Absolutely! Were there any consequences for you from treating yourself the way you did? What were they?

P. In the sense?

I. In the sense.. because of the way you thought about yourself, because of the way you acted towards yourself, were there any impacts to yourself?

P. Yes. I really started looking ugly more ugly than before.

I. You perceived yourself as ugly?

P. Yeah. So all what I thought I was ugly and fat and I wasn’t fat but then I actually became fat. So those negative feelings made me become the person I thought I was.

I. If the same thing happened again, let’s hope not, would you do and think the same things about yourself?.

P. Never in my life.

I. Okay. Why?

P. Because I know what it feels like. And I don’t want to always feel that I should have done this, I should have done this. I want to feel yes I did it. So I want to be like keep thinking.. (unclear).

I. Are there any factors that facilitate or help you to be compassionate towards yourself?

P. Yes. As you said you told you like thought if you do for others, but why not for yourself? So it’s very important to be compassionate to ourselves first of all.

I. So are there any other things in life such as parents, friends or even things such as religion that has helped you to show compassion to yourself more?

P. Yes. My god actually. So whenever I ask my god for something, he has created me not to go and like beg or cry for someone. He he is there with me, so why do I need other people in my life?

I. Yeah. Last question.. Are there any barriers that make it difficult to be compassionate towards yourself?

P. Currently no.. no.

I. No? In the past?

P. Yes. As I said.. umm.. people like.. people call me overconfident when I'm self-compassionate. That affected me. When I helped my friend, they were like you're not the godmother to go and explain people and make them understand, why do you have to worry about them? Many people blame me for supporting her. Many people ignored me, my best friend totally ignored me, she's not even talking to me and that affected me a lot and still it does.

Interview 8.

I. So for the first question…. Can you tell me what the term compassion means to you?

P. Umm.. well, to me it's like understanding, loving, caring and umm.. when it comes to understanding, it's like understanding a person's another person's situation and point of view, loving is like loving unconditionally throughout your flows and caring is like caring whole heartedly. So that's how I see compassion whether it's family, friends or your loved your partner. So that's compassion to me.

I. Ok. Can you talk about your understanding of self-compassion?

P. Self it's like me being compassionate to myself. So Me being compassionate to myself is me being understanding in my own self, when I'm in a really bad situation, trying to bring myself up through worse, it's like talking to a mirror. I want to keep myself strong, keep myself happy, I know what I want.

I. Ok. Brilliant. So..the next set of questions are going to focus on three aspects of compassion. The first one is how we show compassion to other people, the second is how others show compassion to us, and the third is how we are compassionate towards ourselves. So for the first one…I'd like you to think about one or two occasions when a loved one was going through a tough time or difficult situation. This could be a family member or close friend and could be when they were sick, failed an exam, lost a loved one or anything that upset them. Can you tell me if you showed compassion towards them?

P. Umm.. I'll take my fiancé. So, he's a Buddhist and I am a Muslim. So we have like a lot of arguments at home regarding the religion. So.. umm recently his mom fell ill really really ill. And then she was hospitalised and she had to undergo surgery he was.. he's more close towards his mom. So… I was like always there for him and his mom as well. It it was just not only him it's his mom as well and therefore both of them.. I called them day and night. I know that our parents are not okay. But I mean if that's his mom that's my mom as well. So.. that is one incident and there were few other times where I was very helpful to few of my friends which I won't disclose names.

I. Yeah that's okay. And you don't have to tell me the situation as well although I am happy to receive it if you are happy sharing it.

P. So few of my friends when they lost their loved ones and umm.. when they failed a few examinations which I'm still helping them with because they have to resit most of their exams. I also say when at times I wasn't compassionate to some.. I am not like an 100% I would say like a 70%

I. Compassionate to other people?

P. Yes.

I. So.. you said a little bit about why you showed compassion to your fiancé's mother because she is his mother as well, so you feel like you should. But could you tell me if there are other reasons that make you want to show compassion to her or other people who you have shown compassion to?

P. Umm.. Well there were several times where I needed compassion in my life and I did not receive it at those times. Which is why I said I'm kind of selfish when I go to show compassion to some people. If I'm in a really good mood or if I went through the same situation that they're going through, I would just selflessly show compassion to them. So there were times when I needed compassion I did not get any with family or at the time I was dating or even from friends. When I needed them they were like I'm busy.. another time. So.. when I see those people at those hard times, I'm like no I went through the same thing, they shouldn't go through it. So.. I'll do it and then I'll be like satisfied like okay I helped someone. So those that's like the main reason why.

I. Again you answered this a little bit. Can you tell me the things that you did or said to them that made you think you were showing compassion?

P. Phone calls, be there anytime of the day. You wanna talk to me, you just call me I am one call away. I'll wake up and I'll talk to you. And you wanna meet up, yes let's meet up. You wanna talk about it let's talk about it. Come over, me come over to your place.. anything.

I. What were your feelings and thoughts towards them?

P. Umm. At a point I was hurt as much as them because I remembered my past. But at the same time I felt very sorry. Cause I'm sure that I'm not the only person who's going through all these problems, but they are too. But same time I was happy because I was helping them and I know that they have help and they have nothing to worry about. So I kind of secured one part of their worry. So I felt happy and sad.

I. Okay.. And then afterwards…. After you showed compassion, how do you think your words and actions affect them?

P. Umm.. I don't think as a 100% it was perfect. But I did get a thank you for understanding, thank you for calling, thank you for being there for me, like your words helped me a lot, it made me smile. So those yes.

I. How about you? Was there an impact on you by showing compassion?

P. Yes. It made me really happy. And.. I understood that you can't give compassion all the time. Because there might be some people who don't want to give it at all. Cause when it comes to my incident I'm like 50% 50% to be very honest. Sometimes I don't wanna.. compassion because at that time I'm in a bad mood. So when I need compassion I can't give compassion to another person. But at times I can.. So yeah.

I. If the same thing happened again to these people to whom you showed compassion, would you do and say the same things?

P. Umm yes but I will like go through their problems again, see where they went wrong, try to give my opinion. I don't want them to act according to the way I want, but I'll give my opinion. If they feel okay to take it and make sure they don't do it again.

I. Okay.. Are there any factors that facilitate or help you to be compassionate towards others? Just to give a little prompt, it could be circumstances, people in your life, religion, tradition, society, upbringing or anything like that that have helped you to give compassion to others.

P. I'll say experience first. So experience is what… I have experienced… I said when I didn't receive any compassion is one of the main reason why I love to give compassion to other people. And that's one of the reasons why I chose psychology because I have counselling in it. And.. religion religion yes, it helps you to be kind. Religion teaches you to be humble. When it comes to tradition, society not much I'd say. But family, yes. I know my family wasn't compassionate to me at times but I have seen them being compassionate to our relatives or anyone that says we have this problem even financially. I've seen this. So I said that affected a bit but mostly it's experience. What I went through, I don't want another person to go through that. I know how it feels like.

I. Are there any barriers that make it difficult to be compassionate towards others?

P. Umm I'd say people who have done me wrong. It's very hard to for me to show compassion to those people. Like every time they are like oh I have this problem, my head is like you did it to me last time so you deserve it. But at the same time I'm like hmm noo.. okay fine, I'll just talk a bit. But I don't go into deep conversation with them. I'm like ah ok, I'll just listen to them. I wouldn't give my opinion, because if they judge me that way my opinion wouldn't matter. I'll just listen to them. So people who have done me wrong is one. And if I'm not in a good mood, if I'm stressed with studies or something like that, if someone wants compassion even though I give it it's not like I'm 100% it would be just like a 20% of me. So yeah.

I. Great! So you've told me about how you are compassionate towards other people. Now we're going to talk about how others show compassion to you. I am now going to ask you to think about another time – when you were going through a difficult situation or two. Again, this could be when you were sick, failed an exam, lost a loved one or anything that upset you. Can you tell me if anyone showed compassion towards you?

P. I'd say 50% 50%. Because when I was going through a few problems in my life, there were friends who backstabbed me and friends who supported me, there were family members who backstabbed me and there are family members who supported me. Umm my fiancé was very supportive. He's been supportive throughout all my problems so I can't complain about him. I mean he has to be supportive of me (laughs). So.. so yeah they have like umm.. for the friends who supported me a lot, they were very kind enough to at least call me everyday and be like are you okay? Do you want me to listen to anything you have to say? Should I come and visit you, we can take you out. I know the family will be okay, be strong, everything will be okay if you just give it time. So then there were some family members like no it's not going to happen, you just stay like this you should listen to us, you are our child like that.

I. Why do you think some were compassionate and some others were not?

P. The ones who showed compassion actually understood the situation. They are people who actually understand me and they listened to me and they have gone through the same thing. I know that for a fact. Some of my family members have been through the same things that I have gone through. So I know that they understand. But for the people who didn't, I saw it as jealousy, I saw it as not understanding, being very narrow minded, have only one point of view and not being broad minded like at all. Rude I'll say.

I. Okay.. Can you tell me the things that they did or said I mean the people who showed compassion to you that made you feel like they were showing you compassion?

P. Calls. Like I'm there anytime when they need me, they were there for me. If I called them anytime they would just answer me and like yeah tell me, and if I say are you sleeping, no not sleeping tell. They would come over to college to check on me, they would come over to my

place to check on me, and they would just always ask me are you okay? So always stuff like that.

I. What were your feelings and thoughts towards them when they were being compassionate to you?

P. Umm.. happiness. It was very relaxing knowing that okay, apart from the people who don't understand me there are people who actually understand me. So I felt it was like a strength actually. So… if I be like very very honest, it's like the reason I'm strong at this point if I take the very recent incident.. the reason I'm strong at this point is because I know there are people out there who do support me and.. they didn't tell me I'm right nor did they tell me I'm wrong.. they just showed me what can be wrong and what can be right and came to a conclusion and said this is what we think you should. So I would rather have someone show me that way rather than telling me to my face that I'm wrong. So I saw them as a strength.

I. And then afterwards…. How did their words and actions affect you? You said you felt good and it's good to know that.

P. Yeah. I could get back with my studies. And.. thanks to them actually.

I. So you could concentrate on the important things?

P. Concentrate yeah. And I'm taking life smoothly and I won't worry that this is going to happen in future and this barrier will come in the future.. I'm just going with the flow.

I. How about them - How did it make them feel?

P. Umm.. to be very honest, I'm not sure. But for a fact my opinion again not sure at all. I think they felt good. Because there were some of my friends who actually they had never shown compassion to anyone else. But then they were like we were ready to help you, and we are okay, we love to talk to you, so don't be afraid talk to us. So those few friends they have never shown compassion to anyone, so I'm like one of the first few people also, so I guess it made them feel good.

I. Ok, wonderful. If the same thing happened again, would you want them to do and say the same things?

P. Yes, I would love that.

I. Okay. Why?

P. Because they understood. If they understand such the difficult part of that… that difficult part of my life and if I get go through more problems in the future, I would love if they are

there. I know that they would understand. No matter what they would understand. I know for a fact that they would be there for me.

I. Are there any factors that facilitate or help others to be compassionate towards you? Again this could be people, religion, society or anything that may have made it easier for them to show compassion to you?

P. Umm.. I'd say because I was compassionate to them once, and because I've not ignored them when they wanna talk, maybe it's not a problem, maybe it's just they just want to chit chat… I don't even know… Mmm.. I'm a good listener, I've got that from many people. So.. maybe those are the reasons.

I. So those are your personal qualities that make it easier for others to show compassion?

P. Yes.

I. Do you think in general that there are other factors that may have made them compassionate people too?

P. Mmm.. maybe the issues that they went through.. Mmm.. and their family backgrounds. Because there are few of my friends who have really really understanding backgrounds like their parents have called me and asked putha (child) are you okay. So.. I know they have told their parents my problems. There are a few of my friends who share a lot with their moms and they have told me don't worry, hold on. So I'd say family background.

I. Okay.. Are there any barriers that make it difficult for others to be compassionate towards you?

P. Umm.. I'll say at times my family. Because they are strict about who I bring home and who I talk to. So like when they see my mom, they just get this negative vibe. So.. then they're like really scared actually to get involved with my mom. They're like dude your mom will blast us, no way. So I have friends who told that to my face. I can't even complain because that's true. So yeah.

I. We are on to the third aspect now, which is about how we are compassionate towards ourselves. This time I am going to ask you to continue thinking about occasions when you were going through a difficult situation. Do you think that you were compassionate towards yourself?

P. Yes

I. Why do you think that was?

P. Umm.. because at times, at a point where I had no contact with anyone, which I isolated myself. So when I isolated myself I.. I.. actually told my fiancé that I needed space..that's also very less and (unclear).. and he will give me my time and at that time I was umm.. and at that time I was telling like myself okay you have to be strong, this is what you will have to deal with in future, you have to be strong. So I cried my heart out, I… I drew my heart out, I created my heart, I found happiness in (unheard) things..

I. Ok. What were your thoughts and feelings towards yourself?

P. I was really unhappy with myself, I was really sad because I know that I could umm.. get this part of happiness, I'm not reaching it. So I felt very sorry about myself. And.. umm more than sorry I was very angry with myself too. So it was anger and sorry and all that negative feelings.

I. Would you say despite the negative feelings you managed to be compassionate to yourself?

P. Yes

I. How did you treat yourself when you were going through this difficult situation?

P. Umm.. So what I did was I started getting a lot of quotes on strength, and I watched a lot of videos,.. and there's this thing called life quotes on instagram. I used to read them and I used to compare with them they went through this, so I can get through this too. So.. I drew like I said I created stuff. So that's to reduce my stress. So.. continuously I was doing that while doing some studies as well on the process. So mostly encouraging my own self saying do this, and be okay. Because what I wanted to hear from others, when I didn't get that I just gave it to myself. This is what I want to hear from others, they are not going to tell me, so I will give it to myself.

I. Okay. Were there any consequences for you from treating yourself the way you did? What were they?

P. Ummm. I'll say good. Because I wouldn't be even talking to you like if I had not been encouraging my own self, I would be so down and worried about it.. even if someone asked my problem, I am ready to tell. You wanna know my problem, ill tell you my problem. You wanna judge? I don't mind.. so I just give out. I will say I'm stronger now. It's like I've got to a point where I don't give a.. a.. damn I'd say about anyone's judgment. Meet my family, meet my friends, meet my guy. I don't mind. I know what I went through and I know how I encouraged myself. So if anyone tries to question me, I will be like you weren't there for me apart from people who were actually there for me. You weren't there for me so don't judge.

I. Ok.. If the same thing happened again, would you do and think the same things about yourself?

P. Maybe more.

I. More positive?

P. Yeah. Cause if I'm going through the same thing that means I was stupid enough to put my leg back again in the same issue, which should be my fault again. So then I will be like encouraging myself.

I. Are there any factors that facilitate or help you to be compassionate towards yourself?

P. I gain compassion from my loved ones and experience.. like because of those reasons I'm like..

I. So do you take those negative things into a positive way or?

P. I take the negative things in a negative way which takes time for me to process it into positive and then I encourage myself. Like for a very long time I would be like worrying over okay this is wrong, this is wrong, this is wrong and then I will be like no that's not wrong that is right, just do it in this way.

I. Are there any barriers that make it difficult to be compassionate towards yourself?

P. I embrace a lot of negativity. So then it's very hard for me to think and show compassion to myself because I'll be worrying about how the others will (unheard).. at times I rely on others. so when I rely on others and they are not there for me, I'm like.. I'm lost. I just lose hope. But it takes a really long time for me to understand that I don't need others, I have myself, that's it.

I. Okay. That's it.. I'm going to save. First of all, thank you so much

Interview 9.

I. So for the first question…. Can you tell me what the term compassion means to you?

P. Compassion means to me..umm.. it's about feeling empathy towards people who are in umm.. difficult situation.. umm something like that. My family and I, we always tell it to put ourselves in other people's shoes and even if we have to go out of our way to help them, that's what compassion is to me.

I. Can you talk about your understanding of self-compassion?

P. Self-compassion I think is in a way learning how to, not essentially forgive yourself but to have a bit more understanding why you behave yourself like this, or.. yeah I think that's it.

I. Okay. The next set of questions are going to focus on three aspects of compassion. The first one is how we show compassion to other people, the second is how others show compassion to us, and the third is how we are compassionate towards ourselves. So for the first one… I'd like you to think about one or two occasions when a loved one was going through a tough time or difficult situation. This could be a family member or close friend and could be when they were sick, failed an exam, lost a loved one or anything that upset them. Can you tell me if you showed compassion towards them?

P. Yes. Umm.. my father recently.. umm almost a year ago he has suffered a stroke. And yeah he was.. he is okay but the right side of his body isn't a 100% and.. when it happened to him, I kinda thought.. I thought maybe it was cause of his age because he's in his 70s… but umm.. in a way I thought I already know that in the world, these things happen, it's nothing new and happen to anyone. But felt like he is the last person I don't want to say who deserved it okay because I don't believe in that sort of things, things just happen but it was hard and the thing was when this happened umm.. my sister who lives overseas… she's just had a baby so my mother flew over to help her with that and I was living with my father alone and I was at uni when it happened. So, I go home and he said I fell down and I asked are you alright? Yeah everything's fine. Next day he said I have a headache so I took him to the doctor. He said you should have brought.. brought him in yesterday, he had a small stroke. And yeah, I obviously when it is a parents, I think compassion is a thing that is part of the child and the thing what I realised was my father never let it get to him. Umm.. he he just he is just a normal guy who's nice. He's a kind person, he is very he's a quiet kind person. So he didn't even when my mother called from overseas, he didn't want to worry her. And when this happened I didn't even know what a stroke is, I've heard of it but I didn't know what. So yeah I obviously I showed compassion to him but it didn't feel like I was helping helping someone else like another person. Because I'm an extension of him so something like that yeah.

I. Yeah. You don't necessarily have to explain the situation although I'm happy to receive it if you are happy to disclose about the situation. I know you said that this is your father, but could you tell me if there are other reasons as to why you showed compassion to him?

P. Umm.. I think I showed compassion because even from a young age my parents instilled that to me, that those values. Umm.. they said not everybody ummm lives their life, on the same circumstance. People have to go through a lot of things, so we understand it and umm.. whenever you can, you help them even if it is not the most convenient thing to you. So that's why.

I. Can you tell me the things that you did or said to him or other people if there are any multiple occasions you can think of when you showed compassion?

P. See for me, I wasn't aware I'm.. I was deliberately trying to be compassionate. It's just the way I am even when it's not something serious like an illness or anything, I have always felt

like my empathy levels were a bit higher than the average person. It's the first thing that comes to my mind when something happens to someone what would I do if I was you. So.. I wasn't aware that I was showing compassion. But I don't know what I told him, because I was panicking a bit but.. he was in the hospital for a couple of weeks.. so I kinda don't remember what I told him but all I know is that he was glad that I was there.

I. What were your feelings and thoughts towards him or them if you can think of multiple occasions?

P. After the fact?

I. After the fact you showed compassion?

P. Umm.. my feelings towards him.. I felt sorry for him.. umm.. not like in a judgmental way but I felt like I kinda saw myself on that day, because I look like him. So.. I guess in a way I know this is not anything about me but I realised this could this could me in a couple like further down the road. So.. I.. More than more than feeling sorry when this happened I I don't know I didn't know.. I don't know if I was sad because some things are out of our control, but I don't think I had enough time or the awareness to think about my feelings in a way. Because I was more concerned about getting the bills paid and trying to.. umm I'm pretty sure that I didn't even lock the house when I left. So those things were on my mind and so yeah I wasn't aware of that. I was just glad umm.. that the doctor said he's it's not as worse as it could have been.

I. Okay.. That is good news. And then afterwards, after you showed compassion…. How did your words and actions affect them?

P. Because it is my father I guess he would feel like he did a good job raising me. I don't think that's what.. because if I was dad and if I see my son doing these things to me, I would kind of expect him to, right? And.. if I was in his shoes I would be just glad to see.. I think I think when you are sick, the worst thing is to feel isolated and lonely. I think he's just glad I was there and I try to cheer him up cause he likes to watch animal documentaries and all of that so that's what I give him.. I I try to keep him in a good mood, take his mind off all the medicine and tests, the bunch of tests every half and hour or so. Try to keep him like that because that's the way he is.

I. Okay.. okay.. How about you? Was there an impact on you from showing compassion to your father?

P. I don't think so.. Umm.. as I said, it's it's the way I am.. It's it's like it's like (unclear) memory I have. I don't think about it my mom sometimes says you're too compassionate. She thinks I get I get used by who pray on me the way I am. It's not a choice, just happens, can't help it. So yeah I don't..

I. Okay.. If the same thing happens again, let's hope and pray not. But not even this particular situation we are talking about or something like that or if you can think of any other event where you showed compassion to someone, if the same situation happens again, would you do and say the same things?

P. The only thing I think I would do different is, I would be better prepared. But my.. the thoughts, actions I did obviously I can't forget how I will feel if I see the same thing happens to the person or people again. But I think the only thing is I would be better prepared. And… and... So I think the more calm I am, the less nervous I am, the less nervous they will be. So I think yeah I would just be more prepared for the both of us.

I. Are there any factors that facilitate or help you to be compassionate towards others? These could be religion, society, people, the way you were brought up or any other influences.

P. Factors.. I think for me one of the main reasons I am compassionate is I ha.. my father showed me love for animals at a young age and he just said umm.. you know treat them well. He said treat them well and umm.. but what I took from that was they are.. animals are always I feel are they are pure at heart.. in a way because if they don't like you, you know they don't like you. If they like you, you do right? As opposed to a person who would like say something to your face and talk your demise in the back of their head. So this love for animals that he kind of taught me to do, lead me to.. that lead me to seeing a documentary on tv shows about how even carnivore like a lion a predator, always in a way like us looking after their offspring, I saw things like that.. I saw how humans were looking after strange baby animals things like that. So.. I think what I was exposed to as a child always happened to be things of that nature. Because my father you know some men not violent, but they have this tendency to lose their cool. That is not my father. So the.. the other male person I looked up to in the family was not that. He was calm, he was always decent to people, nice so that's what as a child I will have to be that. That's what I looked up to. So whatever compassionate qualities I have I attribute to my parents. Because you know like let's say.. if if a father's immediate reaction to anything is to instigate violence and they have sons, I'm not saying this is a fact but.. they are always more likely to follow their footsteps. So when my parents, I just followed my parents examples basically. When you are a child you just don't know what you observe everything. So I think whatever qualities I have I got from them and what they told me to told me to behave like this, be decent.

I. Okay. Are there any barriers that make it difficult to be compassionate towards others?

P. Sometimes, so.. you mean like if I see someone in a bad situation, something that would stop me from helping them?

I. Yeah, exactly.

P. I think the only thing that would stop me from helping someone like that is if I put my family in danger by doing so. Like let's say.. say when the Tsunami happened, like if I see

someone else struggling and whether I help my my own blood take them into safety or help that person, it's not even a discussion. I think that's the only thing, if my family is in direct harm as a result of helping someone else, it's not going to happen.

I. Ok. Brilliant! So you've told me about how you are compassionate towards other people. Now we're going to talk about how others show compassion to you. So this time, I am now going to ask you to think about another time – when you were going through a difficult situation or two. This could be when you were sick, failed an exam, lost a loved one or anything that upset you. Can you tell me if anyone showed compassion to you?

P. Umm.. yeah. In their own way I guess. See I grew up in another country and when I had to come back it wasn't under the most ideal circumstances. And soon after I landed in Sri Lanka, couple of weeks went by and I fell into this depression where I felt like a rain cloud was following me everywhere. I told my mom I'm feeling like this. But they are old school, born in the 50's in an era when people told to just get over it. So, I told her this is what I was going through and she said why don't you just get over it. I know your normal reaction is angry. I understood, I understand if I grew up in that era. Life is easier for us these days, they had to go through a lot and they just got over it, they had to. And my dad if I like really explain he would understand. But he was already going through some stuff so I didn't want to add to that. Coming from a country where decency and manners are the most important and coming to a country like this where people don't even realise it's missing, I felt helpless. I said I felt like a foreigner in the country I was born. I feel like that now. Nobody understands unless you have been through the same thing. I mean look at the way people drive. So I felt pretty helpless, cause that's the country I grew up in since I was like 12, and then I had to leave. And.. she she knew I was very upset. So whatever things she could do to make me at least a bit okay, she did without questions. So I said I said umm.. even the smallest things that were bothering me.. going back I wouldn't have but and she said what do you sit down and do in front of the computer? I said just check.. umm but that added add that to what I was feeling inside. And I think that okay I don't want to complain but but I get (unclear) she's direct and they helped in the way they know.. like with counselling or going through a professional. Even though that's what I'm studying, I'm scared what I might find out about myself, if I do even at this stage. Even before I told a child psychologist. Coming to that the main reason why I chose psychology, I wanted to fix myself. Umm.. in the first first day of lectures everyone said I want to help other people. But in truth I can't fix others if I'm not.. so my parents, my mom helped. I didn't even tell my dad because I can't. He only has so much going on cause he has been working since he was 19 to.. to like 75. The man is essentially stuck in his own home, cause he can't drive. So I can't possibly add to that. Um… but he can when he sees me he can see something's wrong. Yeah.

I. Okay. So.. seems like you have people who showed compassion to you whether you opened up about it or not. Why do you think people show you compassion besides you are their son in this occasion?

P. So if I take my parents out of the equation, the only other person who has truly shown compassion without any agenda is my best friend growing up over there. He was a Sri

Lankan guy who came to this school. I felt… cause I was the only Sri Lankan in the school for a while, so I felt how lonely I felt. So when this guy came I kinda took him under my own, do this do that.. this is how you do things here and all that.. so he'll understand. So… I can talk to him about anything, he can talk anything with me and he.. I don't know I think the reason he shows compassion to me even more than his own brother is umm.. I figured some people have this goodness in their heart. Some I think most people do but some show compassion for various purposes. But he he has a pure kind heart. And.. he knows I sometimes.. I think that's why. I think the goodness in him I know that's why I think I decided to become his friend in the first place. So yeah I think people show compassion to me not because of the things I have done for them.. but there's there's goodness inside them.

I. Okay. Can you tell me the things that they did or said to you that you felt like were showing compassion?

P. Umm.. well when I say people show compassion to me, I kinda see it as what do they have to gain in by doing this for me and.. there was a time where I was living alone. I was 17-18. My parents have done everything for me up to that point like even washing and cooking and all of that. And they had to leave, my sister had to leave and I was alone. And.. I didn't even have the license to ride my bicycle to go to the grocery store. And he would always say my house.. the door to my house is open, come anytime you want. Anytime (unclear).. and.. that that could be in a way this hospitality that people are taught to behave in such a way, but he didn't have to do that. He had nothing to gain from that. I think I think one of the reasons he showed me compassion was in a way because he remembers what I did for him. Being the only brown guy in a all white school isn't easy, especially when you're a teenager isn't the best time in your life, let's just say. And whenever he was umm.. in a way wondering to an area where he might get into some trouble. Like okay, I got suspended because some guy hit him and I hit back that guy. And and the teacher asked why? He didn't hit you so why did you hit him? This guy you know there was nothing there, for the brown guy.. when I came here for two or three years, I had to go through shit. I'm not going to let that happen to anyone. And I've only known him for a week. He's been in the country for a week. So I think he remembers that and I'm not saying that's the main reason he helped me, but he knows me. I am also selfless, when I feel people are picked on for no reason, I don't like that. I don't like when stuff like that happens to even adults. So he knows that and I think that's the only reason he's good to me, he's essentially the brother I never had. Yeah.. I think there are people who help me not primarily I'm not saying that because some people.. (unclear) but I think it all comes down to empathy. I feel empathy in a different level. I didn't want him to go through anything that I went through. Once you are depressed or once you are going through a really hard time you don't want anyone else to feel remotely feel part of it. So all the racist stuff they said to me about my country and all that I didn't want him to go through that and you know that's the way I am and that's the way people who have received that from me behave towards me.

I. Okay.. What were your feelings and thoughts towards them when they were showing compassion to you?

P. In a way I was glad. I was glad that I was glad that I wasn’t alone in in.. in the sense that I thought from the people I met in my past nobody other than him ever wanted to be genuinely compassionate to me. Like.. you know. From the people I met in my past, I haven’t met even relatives who are truly kind in a way without having an agenda. So when when he’s the only guy outside of my parents who showed me compassion, that’s it. When he was like that I was glad. I was… in a selfish way I was glad I was making the right choice by opening up to him. I don’t open up usually. So when he was like that I was glad and kind of showed me it’s not all bad.

I. Yeah.. And then afterwards after they showed compassion…. How did their words I mean again you answered this a bit. How did their words and actions affect you? Did it change the way you felt at the time when you were going through something difficult?

P. It made a bad situation tolerable. It didn’t change the circumstances, it wouldn’t. But it made the bad circumstances easier to face. That’s what.

I. How about them - How did it make them feel do you think?

P. To help me?

I. Yeah.

P. Umm.. I don’t think I have thought about that.. but I think I really don’t know but.. no I don’t know. I don’t know if they helped because they wanted even a feeling inside. I don’t think that’s him but like I said umm.. umm.. that’s not him. It’s like I think he just did it because it’s a decent thing to do… he in a way knows I don’t open up like that even to my parents. Because I know what they are going through and he also knows what they are going through. so we have an understanding that’s umm.. more than just as of friendship where you just say hi, hello you go and have fun, we talk about stuff. We sometimes sit and talk about meaning of your life and things like that you know. We don’t even sometimes have to speak to guess what we are thinking.. he’s the only other person who understands. So I think he knows I will do that to him in a heart beat and I know that he will do that to me in a heartbeat. So I guess that’s the that’s the reaction we’ve manifested in our friendship.

I. Okay. If you go through the same difficult situation again, would you want him or them to do and say the same things for you?

P. This happened so long ago when I was in high school. I can’t remember how it made me feel. But the brain I had then the brain he had, whatever informs us to take our mind.. I think we were 15 or something. I think both of us did the best of what we know, from whatever we’ve seen in the movies, or whatever our parents have taught us to do in helping in situations like that. So if the umm.. the question was what was the question?

I. If the same thing happens again, if you go through the same situation, would you want the person who showed you compassion to treat you the same way?

P. Yeah. Because as I said.. it it made a unbearable situation tolerable. So what more can you expect from some other person? But even with parents, there's only so much they would feel. They would say they feel.. but only you truly know how deep it is. But yeah it wouldn't make a difference.

I. Are there any factors that facilitate or help them to be compassionate towards you? Could be social factors, personal factors, anything of that sort..

P. Factors.. Umm.. I think the.. I think um what I would I think from what I have learned so far, from what I think it is.. I think it's the way he he.. umm it's the things he's observed from his parents and his older brother. And..I think those influenced him a lot. Because after I came here, he came for a holiday and I met some of the people in school and I don't think they had any.. any influences on the way his psyche works, they were just friends.. circumstantial friends, that's it. And so I think cause I've known most of the things in his head since he moved from Sri Lanka. We were inseparable till the day I had to leave, for 9 years. We grew up together, did everything together.. And he had an uncle. Umm.. he looked up and umm one thing he's had was his relatives that my family and I don't have. His relatives are much more supportive.. they have agendas and those things, but um.. they are a bit more refined in the way it goes.. and when he came to the country even though I was the only brown guy in the school, he didn't feel that isolated I realised because he at any moment he had the support of all his relatives. Because his family was the last to come to Australia, so the relatives had been there for 20-30 years. So they were already established. So he.. he had a huge network but but.. I think those people had an influence in shaping his mind. And because when I choose friends, the only thing I look for, two things I look for.. because they have to match me in a way.. So I think I look for how internally I know them. I can't have a conversation with someone who who would laugh at people that and the kindness, kindness of their heart. I can sense it. So I think that's what.

I. Are there any barriers that make it difficult for others to be compassionate towards you?

P. Yes.. cause as life progresses there are many influences that come in and go. and see for someone to be true to who they are, they have to have a critique, strong mind. Flexibility is important but not when it makes you change who you are in situations. And he's had a few influences that did change him. That's definitely I think the difference between him and I. I am the way I am whether people like me or not. But he will do he will make a lot of conversations to accommodate meaningful relationships. But they have been trying to change him being compassionate to me I think hindered. But never never stopped.. and after the fact he would tell me why this happened and all that. And we were like that. We get.. even if I ask you.. they don't know you, you can't do that. say no say no.. and so we had an understanding for that for a decade. So even though there had been factors that hindered him being compassionate towards me, I don't expect it. But I don't think he owes me anything.. umm you know whenever I'm going through.. he doesn't have to drop everything and help me.. he has to live his own life, you know.. enjoy. Umm.. but it kinda let me down, but that's just my

ego, I wouldn't do... umm.. yeah so people are different so.. but the end of the day there is transparent communication between us.. so whatever happens we will know why.. when people don't know why, they assume things and you never assume you always guess the worst possible thing.

I. We are on to the third aspect now, which is about how we are compassionate towards ourselves. This time I am going to ask you to continue thinking about occasions when you were going through a difficult situation. Do you think that you were compassionate towards yourself?

P. Not initially. Felt like I deserved it. I felt like you are here in this situation because of the decisions you made. Like I believed that. So.. no matter what happens, what bad thing happens, I'm never someone I will always gladly stand in front of a mirror and self-reflect. So.. I think there are stages like that and stages where and then after a while being angry at myself, then I come to a stage where I'm like it's not helping, it's not changing it's only getting worse.. and umm.. then after a while I realise.. I don't know if I've truly ever shown compassion to myself, I haven't forgiven myself for some of the things I have done. But overall I have forgiven myself.. I think me too having self-compassion cause say and my environment the last time I had a drink or smoke that came because I didn't smoke or drink cause I come here I'm depressed, I met a lot of friends.. and so that day haven't since I changed my outlook of things and I'd say that I have forgiven myself for I know I don't do anything bad to other people, that's not who I am so anything bad I don't think I've truly forgiven myself.. cause I've stopped some bad habits like that

I. Again you said a little bit about drinking and then stopping the drinking. Are there any other things that you did to treat yourself whether it's in a negative way or positive way when you were going through something difficult. Did you do other things to treat yourself?

P. I like driving. It's just you and the car. When I'm driving I telling me so one is driving. Another thing is see I had an x-box. When I was in high school I come back I I was depressed and I fell back into that teenage that mind seat. I fell back into find an escape through gaming. Cause I could get lose for like 12 hours. I so driving, that and I must say the drinking was never something that gave me any pleasure, never something I when I wanted to get drunk or anything. The thing drinking did to me was it made me forget for a while. I see I have this messed up memory. I can't remember anything when it comes to like exams but I can remember things I don't forget things I don't forget unimportant things. So drinking was part of that. I knew I'm wasting my life. That's like driving and video games or something.. 80% drinking was like 5% the rest I was just I would just sleep I wouldn't wake up. You wake up and you feel shit. I'm still depressed. But back then it was bad. So just go back to sleep I lock the door my mom says why I would tell her why but she wouldn't understand yeah. yeah

I. Were there any consequences for you from treating yourself the way you did?

P. Treated myself? Ah right. Yeah yeah.. I just umm.. felt like time is the only thing you can't get back. I was aware of the fact I was but I felt like I had no other escape, no other choice. So there's no harm in that's my logic. So that's what I did.

I. If the same thing happened again, would you do and think the same things about yourself?

P. Umm.. if it happens again, I would have learned a lot more about life. Basically so I would know if it does happen again, umm I would I think if it does happen again, it would affect if it happens again I could have a wife and kids and seeing them go through that because of me would kill. So even if it does happen but I don't think because of fall back into bottle. But umm.. I would be better prepared my friends and so yeah I think the only thing I can work for is be better prepared and make make go down umm and another thing I if I go back to drinking I don't want them to see it. I remember seeing my dad drinking when I was like 5. So yeah if it happens again I think I would hope that I would be better prepared.

I. Are there any factors that facilitate or help you to be compassionate towards yourself?

P. Umm.. I truly don't know about this one. I don't know enough to give an honest answer. Or even even a false answer. I don't know enough. I haven't.. See the way I learn things is I usually observe others, how they do things, then I take it on board..how others do it better, how others do it worse.. so I've never seen someone being so self-compassionate towards themselves. I haven't seen that. Maybe I have but it's not in mind. And.. so.. other than that unless I learn it from the classes, I would not know. And at this point in my life, I don't know.

I. It's fine. Last question.. Are there any barriers that get in the way of you becoming compassionate towards yourself?

P. I think low self-esteem. Depression and overweight don't help. I'm not the one to show it. For me to even tell my mom that I was going through depression was huge. I don't tell anyone. And of the barriers, I think that's what low self-esteem. I'm not someone who shows it. I'm not the person to show it. I don't show that I am down. I've always been down in a way but I don't want other people to feel down because of me. I never want to add to anyone's problems. I don't want my parents you know.. and for me to even tell my momma that I was going through depression was huge.. I don't tell anyone. I didn't even tell my friend and he was so mad. Because I.. I know everyone has problems. No matter what socio-economic situation they come from, everybody has problems. And of course I believe that so I don't want to add to anyone's problems. So there is what stops me from feeling self-compassion. And yeah but I think the self-esteem. It's not something umm I don't know I still feel like this I feel that this eminent bad thing that's always about to happen and that's I think that's why in a way I wanted to be supported through... I feel like something bad is going to happen any moment to me or someone I care about. and then I don't how I would react. Umm but I don't know enough to get over this shit..umm..

I. That's the end of the interview. I'm going to save this first.

Interview 10.

I. So for the first question…. Can you tell me what the term compassion means to you?

P. Umm.. so compassion in my understanding is umm being kind to a person, also not also kind treating a person in a very kind way and also empathising to a person's problem, umm.. issue whatever it maybe. Showing an act of kindness towards the hardships that people go through usually.

I. Can you talk about your understanding of self-compassion?

P. Self-compassion.. umm.. so.. my understanding is that umm.. you need to be compassionate umm.. about yourself and show kindness to yourself in order to portrait behaviours to other people. So self-compassion is understanding yourself and trying to do the best for yourself so that you would when you analyse yourself one day or evaluate or go back, think about your actions or something, on a later day that you will be you know fulfilled or satisfied that you have taken correct decisions in your life.

I. The next set of questions are going to focus on three aspects of compassion. The first one is how we show compassion to other people, the second is how others show compassion to us, and the third is how we are compassionate towards ourselves. So for the first one… I'd like you to think about one or two occasions when a loved one was going through a tough time or difficult situation. This could be a family member or close friend and could be when they were sick, failed an exam, lost a loved one or anything that upset them. You don't have to tell me what the situation is, but I want you to think of the situation. Can you tell me if you showed compassion towards them?

P. Yes, I did.

I. Could you tell me why?

P. Because I was able to I was able to relate to that person's situation. I was able to basically empathise to.. empathise because I've undergone a similar situation. So I was able to relate to that person person.. so I feel like I actually I know that I did my level best in terms of showing compassion to that particular person in that particular incident.

I. Can you tell me the things that you did or said to them that made you feel like you were showing compassion?

P. Umm.. I said that I totally understand what they are going through. And I also said that they have the power to not go through it and they have the ability of getting out of that situation. And I also gave them umm.. the option of doing so. Because that person's that

particular decision involved me and some of the people that I know. So I gave the option of getting out of the difficult situation and I also told that..that particular person shouldn't think about what the society would think or what anyone else would think, but solely to think about herself or himself and take that decision. So I feel like I was compassionate enough.

I. What were your feelings and thoughts towards them?

P. I was very I would say I was emotionally down because I was…

I. For them?

P. Yes, for them. I was very emotionally down. It's a memory that I will never be able to erase from my mind because it's not me who went through it, it's that particular person, but I felt really depressed, down and I didn't have an appetite for a couple of days. I felt really helpless and I honestly felt that there was no purpose of living. Umm.. yeah.

I. And then afterwards…. How did your words and actions affect them?

P. I think it helped them get through the situation and they were grateful that I was there for them at that particular time.

I. Was there an impact on you?

P. I felt quite satisfied.. Umm.. more than that I was happy that I was able to.. I was there you know to help them, help that person, to support that person and I felt that I made that person strong, so I felt content. And that was a good feeling that was such a content feeling that I have you know felt for a very long time and I was happy about it.

I. Umm.. Now you said how helping the person did impact you in a positive way in the sense you were satisfied about being able to be there for the person. But also you said when they were going through this, even if this was not you, you too felt depressed and sad about the situation. So if the same thing happened again, knowing that you would go through depressed feelings or the same emotions, would you do and say the same things?

P. Yes I totally would. I wouldn't even think a second or I wouldn't think about it. I would totally go help that particular person out if that particular person is going through the same or different situation.

I. Why?

P. Because one thing if.. because one thing I know that particular person. The second thing is I don't I wouldn't want even an enemy of mine to go through a situation like that. So if I can help that person by talking to her her or him and by supporting that person, I would I

wouldn't think about the impacts that I have, I would also I would solely go and help that person because I know that me talking to that person would actually make a difference.

I. Are there any factors in your life, this could be things, people, experiences that facilitate or help you to be compassionate towards others?

P. Yeah.

I. What are they and can you explain a bit?

P. Umm.. so my father is not my father is not a compassionate person I would say. So he himself.. so when I talk about my father that brings up you know my whole history from the time I remember up to now. So from the time I remember, all I learned and cared about is being kind to others, being good to people, knowing trying to be the best person I am and trying to help people the best way I can. So that was the reason for me to I would say, that was the main reason for me to be this compassionate or kind people person or to be able to relate to a person. That quality came through my experiences in my past.

I. Are there any barriers that make it difficult to be compassionate towards others?

P. No there's nothing like that. I am usually even at work, even at home or wherever I go, if there's an issue I am the first person that someone would come and talk to. Cause I feel like I attract that positive energy and I.. I honestly know that I can relate to a lot of things that people say. I don't make it about me, but I help them and support them by somehow talking to them and strengthening them so that they would be able to get out of that particular situation. But then again as I told you before, I was feeling very down to a point that I felt no purpose of living anymore. If my father was understanding about my struggles, I would have overcome them way easier. But he didn't. I don't blame him either. He's not a compassionate person. But because of the way this society is, people of his generation don't understand these struggles. I mean in this Sri Lankan society, people really don't know much about mental health problems. They just think people with depression are weak and don't really know how to help. Instead they just hide mental illnesses to just avoid judgments.

I. Great! So you've told me about how you are compassionate towards other people. Now we're going to talk about how others show compassion to you. I am now going to ask you to think about another time – when you were going through a difficult situation or two. This could be when you were sick, failed an exam, lost a loved one or anything that upset you. Can you tell me if anyone showed compassion towards you?

P. Yes there are many people that have showed compassion to me.

I. Why do you think that was?

P. Umm.. some people solely because they cared about me. And some were because umm.. I feel I felt like they were you know they felt like they were obliged to understand and help me out. But most people in my life had been have been compassionate to me because they solely cared about me.

I. Okay.. Can you tell me the things some of the things that they did or said to you which made you think they were showing compassion?

P. Umm.. they constantly supported me. They called and followed up on my wellbeing. And they also told me that no matter what, there's this thing that one person said to me that really struck me. That I shouldn't let anyone's words actions affect me unless I let it affect myself. So that.. so that I should be in control of my emotions and what I need to process and what I shouldn't. So those words of kindness, those words that actually brought me ..brought.. actually made me a strong person you know the person I am. And I feel through those terrible hardships that I have gone through in life I am who I am is because of those.

I. When they were sowing compassion.. What were your feelings and thoughts towards them?

P. I was very grateful that I have people like that in my life. And I also knew that no matter what no matter what happens, that those people will always be there with me to strengthen me and that I could run to them at anytime at any hour at any time and I I always knew that they would constantly console me and uplift my negativity.. So.

I. I think you already answered the next question. But I expect a bit more information.. And then afterwards…. How did their words and actions affect you?

P. I was able to get through it very.. I would say I took the least amount of time to get through it because I had those people positively influencing my actions, my decisions and helping me out and always following up on me. So that was umm.. so I was able to get through it very quickly. So I felt less pain I would say and if those people were not in my life I don't think I would have gotten through it as easily as I did.

I. How do you think helping or showing compassion to you made them feel?

P. I'm sure they felt good about themselves too. They've been through similar issues. More than helping me, they were honestly doing themselves a favour. Because something that came to their mind was when they went through it they didn't have anyone, but when a close friend of theirs that's me when I go through it and they knew that they could help me, I'm sure they felt the same contentment I feel when I help someone else in their hardships.

I. If the same thing happened again, would you want them to do and say the same things?

P. Yes definitely. Because not the same things but I would want them to show same or more of compassion and kindness and strengthen me because they positively influence. They are a positive influence in my life. So I would always want those particular people to help me and uplift me.

I. Okay. Are there any factors that facilitate or help others to be compassionate towards you?

P. Yeah it could be their upbringing, could be their education, paths that they have chosen in their life, their experiences.. yeah.

I. Do you think you as a person has any impact on them wanting to show compassion to you or does it not matter who they are showing compassion to?

P. No I would totally say that they would show compassion to anyone they meet because of their qualities, their personalities etc.

I. Are there any barriers that make it difficult for others to be compassionate towards you?)

P. I don't think there are any barriers.

I. We are on to the third aspect now, which is about how we are compassionate towards ourselves. This time again I want you to continue thinking about occasions when you were going through a difficult situation. Do you think that you were compassionate towards yourself?

P. Umm.. yes I did. Actually I have mixed feelings about it. Sometimes I feel like no I didn't consider myself.. I should have had more positive-ness over that situation without you know getting so negative and getting influenced by that particular action. But also I feel like I was compassionate towards myself. So yeah I have mixed feelings about that particular situation and how I was being compassionate towards it.

I. Why do you think at times you were being compassionate and at times you weren't?

P. That was.. this particular incident happened sometime back. So now when I think about it and I know that I could have done something more. So then I felt like I wasn't being compassionate enough for myself. So yeah that's because I've grown out of it and now when I look back and now that I understand the context and why that particular incident happened and how it happened and the reason of you know all of that. The reason through all that negativity that entire you know uncomfortable situation. Now that I know the reason behind it I feel like I would.. then if I had a better understanding about the particular situation, I would have had the ability to be more compassionate about myself.

I. What were your thoughts and feelings towards yourself when you were going through this?

P. Umm.. I regretted. First I thought that it is my mistake. But then later on with time I understood that it just happens and it’s not only me who went through it. But a lot of people go through similar issues like that and that it’s okay to you know go through it and I.. I perceived it as a learning experience for myself. So.. yeah.

I. How did you treat yourself?

P. Umm.. not good at all. I was.. like I said before I was pretty down. And I.. I didn’t self harm. But I was having thoughts of you know I had suicidal thoughts, but I.. I was I would say I was not gutty enough to you know go ahead with that. But if I was a person who would harm myself, I would have definitely gone to it, done it. But umm.. I was in the verge of doing it but I never had the guts to do it. I was feeling very down and I just didn’t know how to get out of it. That was the worst feeling ever.

I. Well I am very sorry to hear that.. Were there any consequences for you from treating yourself the way you did?

P. As in like

I. Yeah did it have any impact on you?

P. Yeah yes. Yeah.. I.. I felt like my performance as.. So in that particular.. that particular incident was.. how can I say it.. This incident that I am relating to impacted part of my life-style. So I felt like me being able to contribute to that particular society was hindered by me not being strong enough and if I had seen that situation in a different way I would have been able to contribute more and be a different person in that particular society.

I. Okay.. Umm.. If the same thing happened again, would you do and think the same things about yourself?

P. As in if the same thing happens again to me, would I act the same way?

I. Yeah.

P. Never, I wouldn’t. Because I’ve evolved with.. now that I know that I’ve gotten through that experience I know how to deal with it. And I feel like as a person I’ve evolved and I’ve learned so much about things like that so that you know as a person I’m ready to face similar things and not get hurt about it. I have more self-confidence in me than I have before in the past.

I. Are there any factors in your life such as people, religion, society, upbringing, culture anything like that, that facilitate or help you to be compassionate towards yourself?

P. Umm.. people yes, yes. People did. Umm.. religion.. Culture I wouldn't say so. But people definitely, there are certain people that you know you look up to when you know that they are your you know figures of like you respect them. They are your role models to be a good person. Umm.. yeah religion too. But I don't solely depend as in I don't think that religion is the only way for a person to be compassionate. I know that religion teaches all these good things about it. But it's how you perceive it and it's how you.. you know also there are people who are very religious but are not compassionate.. so it's how you perceive it.. it's how you let everything else affect your actions. So yeah.

I. Are there any barriers that make it difficult to be compassionate towards yourself?

P. My father I think. Actually the society. Remember how I told you how sad I was before. I was feeling very down to a point that I felt no purpose of living anymore. If my father was understanding, I would have overcome way easier. I don't blame him. He's not a compassionate person. But because of the way this society is, people of his generation don't understand these struggles. In Sri Lanka, people don't really know much about mental health. They just think people with depression are weak and don't know how to help. Instead, they just hide mental illnesses to just avoid judgements

I. Yeah. That's a shame.
